# Computational Analysis of the Mode of Action of Disopyramide and Quinidine on hERG-Linked Short QT Syndrome in Human Ventricles

**DOI:** 10.3389/fphys.2017.00759

**Published:** 2017-10-04

**Authors:** Dominic G. Whittaker, Haibo Ni, Alan P. Benson, Jules C. Hancox, Henggui Zhang

**Affiliations:** ^1^Biological Physics Group, School of Physics and Astronomy, University of Manchester, Manchester, United Kingdom; ^2^School of Biomedical Sciences, University of Leeds, Leeds, United Kingdom; ^3^Multidisciplinary Cardiovascular Research Centre, University of Leeds, Leeds, United Kingdom; ^4^School of Physiology, Pharmacology and Neuroscience, Cardiovascular Research Laboratories, School of Medical Sciences, University of Bristol, Bristol, United Kingdom; ^5^School of Computer Science and Technology, Harbin Institute of Technology, Harbin, China; ^6^Space Institute of Southern China, Shenzhen, China

**Keywords:** arrhythmia, short QT syndrome, drug modeling, potassium channels, human ventricles, class 1a anti-arrhythmics

## Abstract

The short QT syndrome (SQTS) is a rare cardiac disorder associated with arrhythmias and sudden death. Gain-of-function mutations to potassium channels mediating the rapid delayed rectifier current, *I*_Kr_, underlie SQTS variant 1 (SQT1), in which treatment with Na^+^ and K^+^ channel blocking class Ia anti-arrhythmic agents has demonstrated some efficacy. This study used computational modeling to gain mechanistic insights into the actions of two such drugs, disopyramide and quinidine, in the setting of SQT1. The O'Hara-Rudy (ORd) human ventricle model was modified to incorporate a Markov chain formulation of *I*_Kr_ describing wild type (WT) and SQT1 mutant conditions. Effects of multi-channel block by disopyramide and quinidine, including binding kinetics and altered potency of *I*_Kr/hERG_ channel block in SQT1 and state-dependent block of sodium channels, were simulated on action potential and multicellular tissue models. A one-dimensional (1D) transmural ventricular strand model was used to assess prolongation of the QT interval, effective refractory period (ERP), and re-entry wavelength (WL) by both drugs. Dynamics of re-entrant excitation waves were investigated using a 3D human left ventricular wedge model. In the setting of SQT1, disopyramide, and quinidine both produced a dose-dependent prolongation in (i) the QT interval, which was primarily due to *I*_Kr_ block, and (ii) the ERP, which was mediated by a synergistic combination of *I*_Kr_ and *I*_Na_ block. Over the same range of concentrations quinidine was more effective in restoring the QT interval, due to more potent block of *I*_Kr_. Both drugs demonstrated an anti-arrhythmic increase in the WL of re-entrant circuits. In the 3D wedge, disopyramide and quinidine at clinically-relevant concentrations decreased the dominant frequency of re-entrant excitations and exhibited anti-fibrillatory effects; preventing formation of multiple, chaotic wavelets which developed in SQT1, and could terminate arrhythmias. This computational modeling study provides novel insights into the clinical efficacy of disopyramide and quinidine in the setting of SQT1; it also dissects ionic mechanisms underlying QT and ERP prolongation. Our findings show that both drugs demonstrate efficacy in reversing the SQT1 phenotype, and indicate that disopyramide warrants further investigation as an alternative to quinidine in the treatment of SQT1.

## Introduction

The short QT syndrome (SQTS) is a genetic condition in which the QT interval on the ECG is abnormally short, leading to increased risk of atrial and/or ventricular arrhythmias and sudden cardiac death (SCD; Schimpf et al., 2005). The SQTS is genetically heterogeneous, with a complex genotype-phenotype relationship (Harrell et al., 2015). The first identified form of the SQTS (SQT1) was caused by a missense mutation (N588K) to the human *Ether-à-go-go-Related Gene* (*hERG*) encoding the α subunit of channels carrying the rapid delayed rectifier potassium current, *I*_Kr_ (Brugada et al., 2004). At physiological temperature, the N588K-hERG mutation has been shown to significantly attenuate inactivation, without altering the voltage dependence of activation (McPate et al., 2005), causing a “gain-of-function” in *I*_Kr_ which significantly reduces the QT interval (QTc ≤ 300 ms; Brugada et al., 2004).

The current frontline treatment for SQTS patients is use of an implantable cardioverter-defibrillator (ICD) device, which protects against sudden arrhythmic death (Giustetto et al., 2006). However, T-wave oversensing, which leads to erroneous identification of tachyarrhythmic events, can be an issue with such devices, as T-waves often appear tall and peaked in SQTS patients, necessitating device reprogramming (Schimpf et al., 2005). Furthermore, ICDs are not particularly suited to some pediatric patients (Villafañe et al., 2013), necessitating the pursuance of alternative, pharmacological approaches. Data on SQTS patients are comparatively sparse, due to the rarity of the condition. However, several studies have reported on the effectiveness of quinidine at restoring the QT interval and ventricular effective refractory period (ERP) in the setting of the SQTS (Gaita et al., 2004; Wolpert et al., 2005; Giustetto et al., 2006; Hu et al., 2017), as well as on the lack of effect of other hERG inhibitors such as sotalol, ibutilide, and flecainide (Gaita et al., 2004; Giustetto et al., 2015). A few patient studies have also shown that disopyramide has some efficacy in reversing the SQTS phenotype (Schimpf et al., 2007; Mizobuchi et al., 2008; Giustetto et al., 2011).

Detailed *in vitro* studies into the pharmacology of N588K-hERG linked SQT1 (McPate et al., 2006, 2008) used whole-cell patch clamp measurements of expressed *I*_hERG_ at 37°C to assess the blocking potency of several canonical hERG inhibitors on N588K mutant hERG channels. In those studies disopyramide emerged as a potential alternative to quinidine, which is more commonly used in SQT1 (Gaita et al., 2004; Giustetto et al., 2006; Hu et al., 2017), as the IC_50_ (half maximal inhibitory concentration) was increased only 1.5-fold compared to wild type (WT) hERG channels (compared to a 3.5-fold increase reported for quinidine). The reason for the comparative effectiveness of these two agents appears to be due to the fact that neither drug strongly relies on hERG channel inactivation gating in order to exert an inhibitory effect (McPate et al., 2006, 2008; Perrin et al., 2008).

The underlying mechanisms by which combined ion channel blocking actions of disopyramide and quinidine exert anti-arrhythmic effects in the setting of SQT1 are not well understood. Whereas, several studies have previously used computer models to gain insights into QT interval shortening and pro-arrhythmic effects of SQT1 mutant hERG channels in human ventricles (Zhang and Hancox, 2004; Weiss et al., 2005; Adeniran et al., 2011), significantly less is known about the mode of action of pharmacological agents on human ventricular electrophysiology in SQT1. A recent simulation study (Luo et al., 2017) adopted a simplified “pore block” approach, with one-dimensional (1D) and 2D tissue simulations, to investigate effects of quinidine and disopyramide in the setting of SQT1, but failed to replicate beneficial effects of disopyramide seen in the clinical setting (Schimpf et al., 2007; Mizobuchi et al., 2008; Giustetto et al., 2011). The present study was undertaken to provide comprehensive information regarding the actions of both quinidine and disopyramide in the setting of N588K-linked SQT1, incorporating drug binding kinetics and 3D tissue simulations.

## Methods

### Model development

The O'Hara-Rudy dynamic (ORd) model of the human ventricular action potential (AP; O'Hara et al., 2011) was used for simulations in this study, due to its extensive experimental validation and ability to reproduce complex behaviors such as early after depolarizations (EADs)—a crucial requirement when simulating pharmacological agents which pose a torsadogenic risk. An updated form of the ORd model described recently (Mann et al., 2016) was used, as this configuration gave a QT interval shortening which was more concordant with clinical observations, and reproduced increased T wave amplitude observed in the SQTS (Schimpf et al., 2005; Anttonen et al., 2009). Furthermore, this allowed comparative investigations with an updated form of the 2006 ten Tusscher et al. (TP) model (ten Tusscher and Panfilov, 2006) in order to assess model dependence of results (see Discussion and Supplementary Material Section 1.3).

The ORd model was further modified by (i) replacing the fast sodium current, *I*_Na_, formulation with that of the Luo-Rudy model (Luo and Rudy, 1994) to facilitate propagation in tissue (see Supplementary Material Section 1.3 for further consideration of this point), and (ii) implementing a Markov chain formulation of *I*_Kr_. Rate transitions of the drug-free *I*_Kr_ Markov model describing WT and the SQT1 mutant N588K were updated from our previous study (Adeniran et al., 2011) and validated using voltage and AP clamp experimental data conducted at 37°C (McPate et al., 2005, 2009) to better describe kinetic changes accounting for impaired inactivation during the time course of the AP. New fits to experimental data and kinetic parameters are given in Figure S1 and Table S1, respectively. As SQTS mutations are expressed heterozygously *in vivo*, we constructed a heterozygous formulation (WT-N588K) consisting of 50% WT and 50% N588K channels. This approach has been adopted in a previous investigation of SQT1 (Loewe et al., 2014) and the heterozygote formulation, which is used throughout the study, is hereinafter referred to simply as the SQT1 condition.

### Modeling actions of disopyramide and quinidine on hERG channels

**Figure 1:**
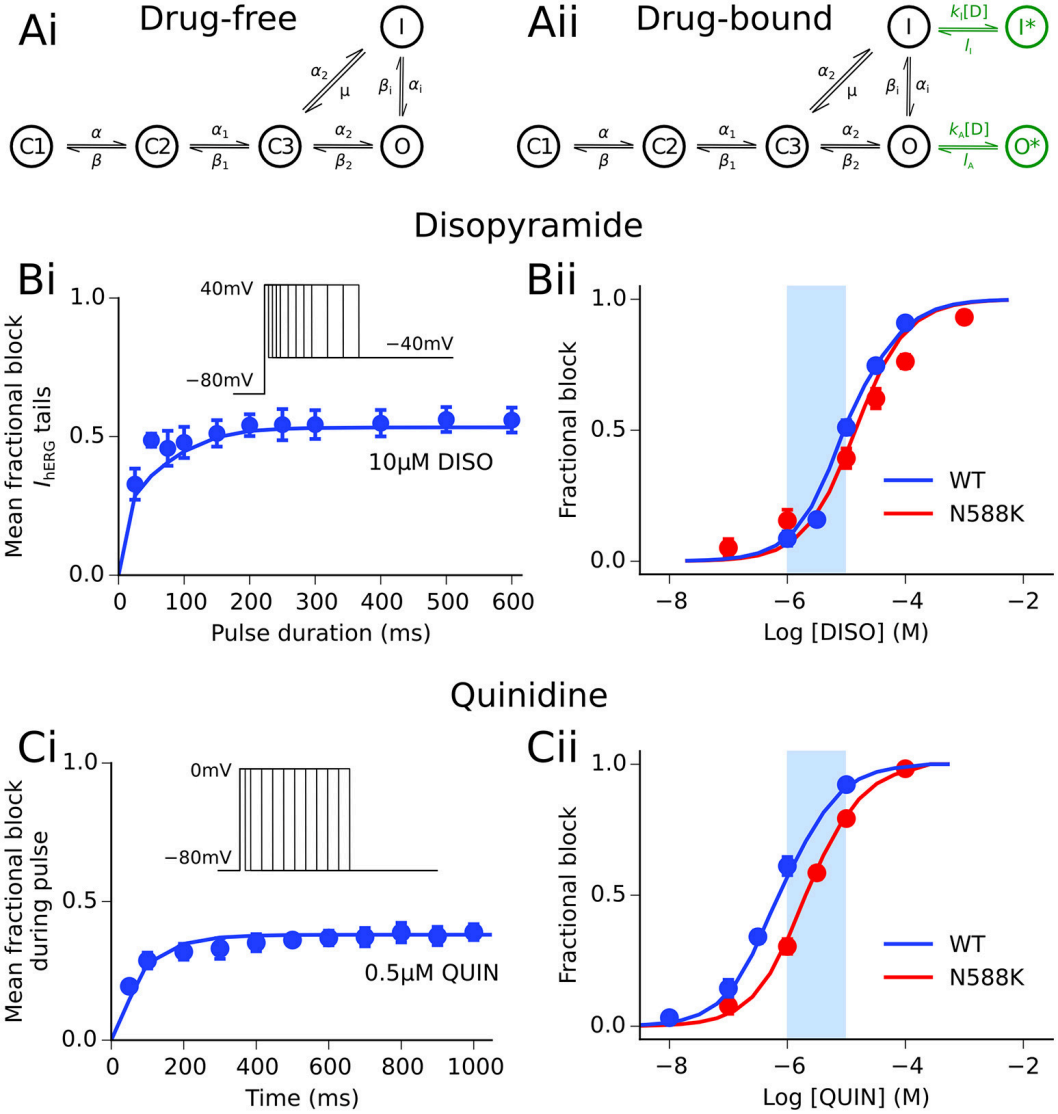
Disopyramide and quinidine interactions with hERG channels. **(Ai)** Drug-free and **(Aii)** drug-bound (additional states shown in green) Markov chain models of *I*_Kr/hERG_. Simulated (solid line) and experimental (points) mean fractional block by disopyramide (DISO) of *I*_hERG_ tail currents following pulse protocol (shown inset) **(Bi)**, and dose-response curve under WT (blue) and SQT1 mutant N588K (red) conditions **(Bii)**, where IC_50_ values are 10.77 and 15.77 μM, respectively. Simulated (solid line) and experimental (points) mean fractional block by quinidine (QUIN) of *I*_hERG_ during pulse protocol (shown inset) **(Ci)**, and dose-response curve under WT (blue) and SQT1 mutant N588K (red) conditions **(Cii)**, where IC_50_ values are 620 nm and 2.16 μM, respectively. Experimental data at 37°C are taken from Paul et al. (2001, 2002) and McPate et al. (2008). The light blue shaded area shown for reference in panels **(Bii,Cii)** corresponds to the concentration range of 1–10 μM.

Disopyramide and quinidine are class Ia agents, i.e., drugs which exert an anti-arrhythmic effect through block of the fast sodium current as well as repolarizing K^+^ currents (Roden, 2014). In order to simulate interactions between both drugs and the hERG/*I*_Kr_ channel, the Markov chain model of *I*_Kr_ was extended to include a drug-bound open and drug-bound inactivated state, as described previously (Perrin et al., 2008), shown in Figure 1. The formulation for *I*_Kr_ is given by:

(1) $$\begin{array}{l} I_{\text{Kr}}=g_{\text{Kr}}O\left(V-E_{\text{Kr}}\right), \end{array}$$

(2) $$\begin{array}{l} \frac{\text{d}C1}{\text{d}t}=\beta C2-\alpha C1, \end{array}$$

(3) $$\begin{array}{l} \frac{\text{d}C2}{\text{d}t}=\alpha C1\;+\;\beta_{1}C3-\left(\beta +\alpha_{1}\right)C2, \end{array}$$

(4) $$\begin{array}{l} \frac{\text{d}C3}{\text{d}t}=\alpha_{1}C2\;+\;\beta_{2}O+\mu I-\left(\beta_{1}+{2\alpha }_{2}\right)C3, \end{array}$$

(5) $$\begin{array}{l} \frac{\text{d}I}{\text{d}t}=\alpha_{2}C3\;+\;\beta_{i}O+l_{I}I^{*}-\left(\mu +\alpha_{i}+k_{I}\right)I, \end{array}$$

(6) $$\begin{array}{l} \frac{\text{d}O}{\text{d}t}=\alpha_{2}C3\;+\;\alpha_{i}I+l_{A}O^{*}-\left(\beta_{2}+\beta_{i}+k_{A}\right)O, \end{array}$$

(7) $$\begin{array}{l} \frac{\text{d}I^{*}}{\text{d}t}=k_{I}\left[D\right]I-l_{I}I^{*}, \end{array}$$

(8) $$\begin{array}{l} \frac{\text{d}O^{*}}{\text{d}t}=k_{A}\left[D\right]O-l_{A}O^{*}, \end{array}$$

where *g*_Kr_ is the maximal channel conductance, *O* an open state, *I* an inactivated state, *C1, C2*, and *C3* are closed states, O^*^ and I^*^ represent drug-bound open and inactivated states, respectively, *V* is the transmembrane voltage, *E*_Kr_ is the K^+^ reversal potential, *k*_X_ and *l*_X_ are binding and unbinding rate constants for states of type X, respectively, and [*D*] is the drug concentration. Rate transitions to drug-bound states describing the concentration- and state-dependent block of hERG channels by disopyramide and quinidine were based on prior recordings from our laboratory made at 37°C (Paul et al., 2001, 2002). Details of parameterization of the drug-bound Markov chain *I*_Kr_ model to experimental data are given in the Supplementary Material, and parameters are detailed in Table S2.

### Modeling actions of disopyramide and quinidine on sodium channels

**Figure 2:**
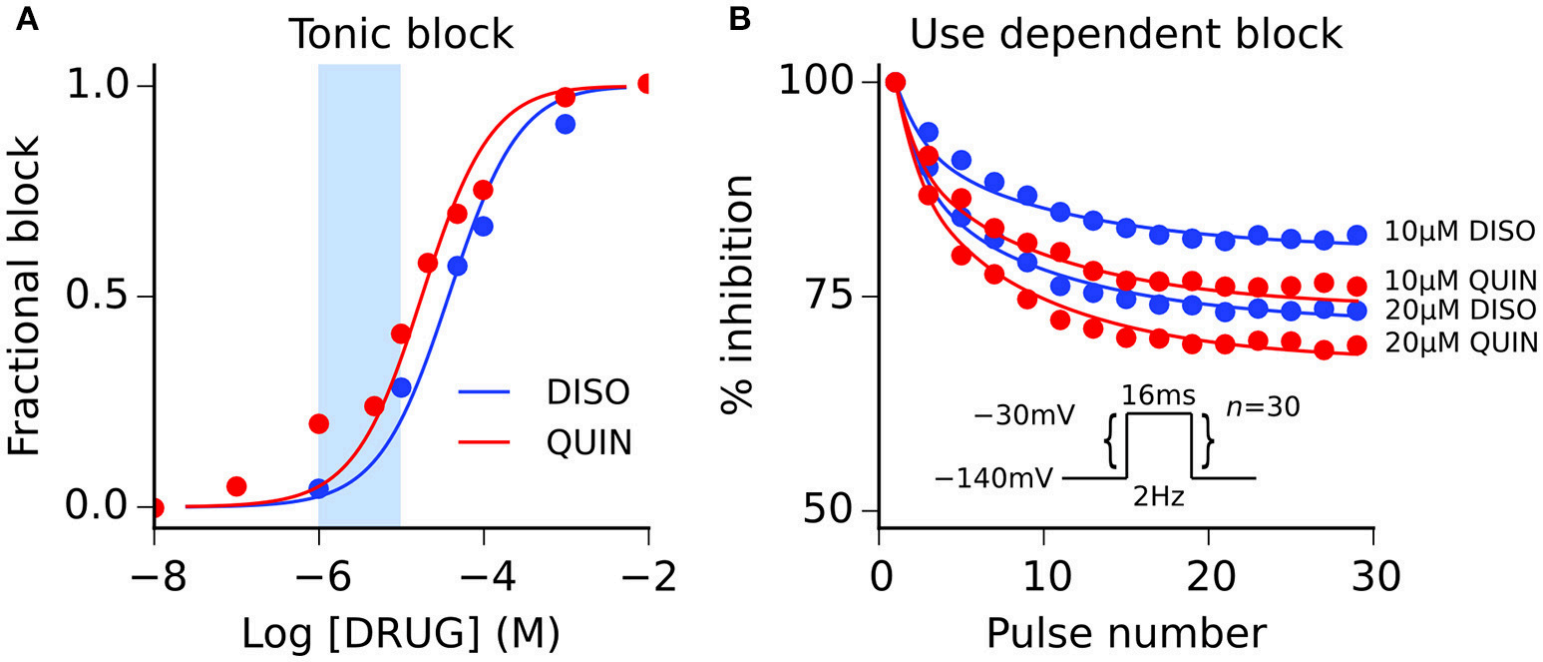
Disopyramide and quinidine interactions with sodium channels. **(A)** Simulated (solid lines) and experimental (points) tonic block of sodium channels by disopyramide (DISO) and quinidine (QUIN), and **(B)** use-dependent block of sodium channels elicited using pulse protocol (*n* = 30 pulses) shown inset. Experimental data are taken from Koumi et al. (1992), where tonic block IC_50_ values are 36 and 17 μM for disopyramide and quinidine, respectively. The light blue shaded area shown for reference in panel **(A)** corresponds to the concentration range of 1–10 μM.

Both disopyramide and quinidine have been shown previously to exhibit use dependent block of *I*_Na_ (Koumi et al., 1992), with quinidine producing more potent tonic block (i.e., resting and inactivated channel block) than disopyramide. Interactions between both drugs and sodium channels were represented using the guarded receptor formalism (Starmer et al., 1984), in which drugs bind to ion channel conformations with constant affinity but access to each binding site is “guarded” by the state of the ion channel. Disopyramide and quinidine were assumed to bind to activated, inactivated, and resting sodium channels based on experimental evidence (Koumi et al., 1992), with the formulation for *I*_Na_ given by:

(9) $$\begin{array}{l} I_{\text{Na}}=g_{\text{Na}}\left(1-b_{A}-b_{I}-b_{R}\right)m^{3}hj\left(V-E_{\text{Na}}\right), \end{array}$$

(10) $$\begin{array}{l} \frac{\text{d}b_{A}}{\text{d}t}=k_{A}\left[D\right]m^{3}hj\left(1-b_{A}-b_{I}-b_{R}\right)-l_{A}b_{A}, \end{array}$$

(11) $$\begin{array}{l} \frac{\text{d}b_{I}}{\text{d}t}=k_{I}\left[D\right]\left(1-hj\right)\left(1-b_{A}-b_{I}-b_{R}\right)-l_{I}b_{I}, \end{array}$$

(12) $$\begin{array}{l} \frac{\text{d}b_{R}}{\text{d}t}=k_{R}\left[D\right]\left(1-m^{3}\right)hj\left(1-b_{A}-b_{I}-b_{R}\right)-l_{R}b_{R}, \end{array}$$

where *g*_Na_ is the maximal channel conductance, *b*_A_, *b*_I_, and *b*_R_ represent the fractional block of activated, inactivated, and resting states, respectively, *m* is the activation gate, *h* and *j* are the fast and slow inactivation gates of the sodium channel, respectively, *E*_Na_ is the Na^+^ reversal potential, and all other parameters retain their previous definitions. Model fits to experimental data on the dose-dependence of tonic block and development of use-dependent block are shown in Figure 2, and drug binding parameters are given in Table S3. Parameterization of all ion channel drug interaction models was performed using a bounded Nelder-Mead simplex algorithm (Moreno et al., 2016); more details are given in the Supplementary Material.

### Modeling actions of disopyramide and quinidine on other channels

In addition to *I*_Na_ and *I*_Kr_ block, disopyramide and quinidine exert secondary, generally weaker actions on various other ion channel currents; namely, the L-type calcium current, *I*_CaL_, the transient outward potassium current, *I*_to_, the slow delayed rectifier potassium current, *I*_Ks_, the inward rectifier potassium current, *I*_K1_ (quinidine only), and the late sodium current, *I*_NaL_ (quinidine only). For these other ionic substrates affected, simple pore blocks were simulated based on published dose-response curves. Within the framework of pore block theory (Brennan et al., 2009), the maximal conductance *g*_*i*_ of an ionic current type *i* is modified in a concentration-dependent manner, such that

(13) $$\begin{array}{l} g_{i}=g_{control,i}\frac{1}{1+{\left({\left[{\text{IC}}_{50}\right]}_{i}/\left[D\right]\right)}^{nH}}, \end{array}$$

where *g*_control, i_ represents the maximal conductance of the *i* channel in drug-free conditions and *nH* is the Hill coefficient. IC_50_ values extracted from the literature for disopyramide and quinidine are given in Table 1. Comparative IC_50_ values for block of *I*_Kr_ and *I*_Na_ (tonic block) by both drugs are given in legends for Figures 1, 2.

**Table 1:** Secondary pharmacological effects of disopyramide and quinidine on ion channels.

	**Disopyramide**		**Quinidine**	
	**IC_50_ (μM)**	**Source**	**IC_50_ (μM)**	**Source**
***I***_CaL_	1036.7	Kramer et al., 2013	14.9	Zhang and Hancox, 2002
***I***_to_	20.9	Hanada et al., 2003	21.8	Nenov et al., 1998
***I***_Ks_	88.1	Satoh, 2000	44.0	Kang et al., 2001
***I***_K1_	–	–	42.6	Nenov et al., 1998
***I***_NaL_	–	–	12.0	Wu et al., 2008

*A summary of half maximal inhibitory concentration values (IC_50_) extracted from the literature*.

The therapeutic steady-state plasma levels of both disopyramide and quinidine are reported to be ~2–5 μg/ml (Roden and Woosley, 1983), which corresponds to a concentration range of ~6–15 μM for both agents. However, actual bioavailability *in vivo* is less than this due to pharmacokinetic factors such as plasma protein binding. 1 and 2 μM of disopyramide and quinidine likely constitute realistic maximal unbound concentrations (Sagawa et al., 1997). To encompass likely total as well as unbound concentrations, we elected to simulate effects of a wide range of concentrations of both agents (0.2–20 μM) at the single cell level, and a narrower set of more “clinically-relevant” concentrations (1, 2, 5, 10 μM) at the tissue level (represented by light blue shaded regions on dose-response curves in Figures 1, 2).

### Tissue simulations

The monodomain equation (Clayton et al., 2011) was used to describe the propagation of APs in tissue:

(14) $$\begin{array}{l} \frac{\partial V}{\partial t}=\nabla \left(\text{D}\nabla V\right)-\frac{I_{\text{ion}}}{C_{\text{m}}}, \end{array}$$

where **D** is the diffusion coefficient tensor, *I*_ion_ is the total ionic current, and *C*_m_ is the membrane potential. Equation (14) was solved numerically using a finite-difference PDE solver based on the explicit forward Euler method, as described previously (Whittaker et al., 2017). The pseudo-ECG (pECG) was calculated according to (Plonsey and Barr, 2013), i.e.,

(15) $$\begin{array}{l} \Phi (x\prime ,y\prime ,z\prime )=\int^{\;}(-\nabla V)•\left[\nabla \frac{1}{r}\right]d\Omega , \end{array}$$

(16) $$\begin{array}{l} r={\left[{\left(x-x\prime \right)}^{2}+{\left(y-y\prime \right)}^{2}+{\left(z-z\prime \right)}^{2}\right]}^{\frac{1}{2}}, \end{array}$$

where Φ is a unipolar potential generated by the multicellular tissue preparation, *r* is the distance between a source point (*x, y, z*) and the coordinate of a virtual electrode (*x*′, *y*′, *z*′), and Ω is the domain of integration.

### Heterogeneous 1D strand model

A 1D transmural model of human ventricle comprising 25 endocardial (ENDO) cells, 35 mid-myocardial (MCELL) cells, and 40 epicardial (EPI) cells was used, with a total length of 15 mm as in our previous studies (Adeniran et al., 2011, 2017). Conduction was isotropic, except for a five-fold decrease in **D** at the border of the MCELL and EPI regions (Gima and Rudy, 2002; Zhang and Hancox, 2004; Adeniran et al., 2011, 2017). Planar waves were initiated by applying a stimulus at the endocardial surface, which propagated transmurally along the fiber toward the epicardial surface.

For the 1D pECG, the virtual electrode was placed 2.0 cm away from the epicardial end of the fiber, and the end of the T wave was defined as the intersection of the steepest portion of the descending limb with the baseline (Gima and Rudy, 2002). As in our previous study of SQT1 (Adeniran et al., 2011), the ENDO:EPI:MCELL ratio of *I*_Kr_ maximal conductance was adjusted to 1.0:1.6:1.0, based on experimental measurements of transmural hERG mRNA expression (Szabó et al., 2005). This resulted in larger T wave amplitude in the SQT1 condition—a hallmark of SQTS patients (Schimpf et al., 2005; Anttonen et al., 2009).

### Heterogeneous 3D left ventricular wedge model

A 3D wedge model of the left ventricular free wall incorporating fiber and sheet orientations taken from a DT-MRI scan of a human heart (Benson et al., 2011) was used to assess the anti-arrhythmic potential of disopyramide and quinidine in the setting of re-entrant excitation waves in SQT1. The tissue geometry is segmented into ENDO, EPI, and MCELL regions as described previously (Benson et al., 2011), and is shown in Figure S2. Further details regarding conductivities and orthotropy ratio can be found in the Supplementary Material. Re-entry was initiated using the phase distribution method (Biktashev and Holden, 1998; Colman et al., 2017; Whittaker et al., 2017), in which an artificial asymmetric conduction pattern is created, which develops into a re-entrant scroll wave. Multiple initial condition phase maps were used (*n* = 6) for 3D re-entry simulations (see Figure S3). The lifespan of re-entry was calculated based on time domain signals taken from transmural APs. Frequency domain signals were obtained through Fourier transform analysis of pECGs and used to compute the dominant frequency (DF) based on the largest peak in the power spectrum density, as described previously (Whittaker et al., 2017). The virtual electrode for recording the pECG was placed ~3.0 cm away from the center of the endocardial surface of the wedge, as illustrated in Figure S6.

## Results

### Single cell investigations

**Figure 3:**
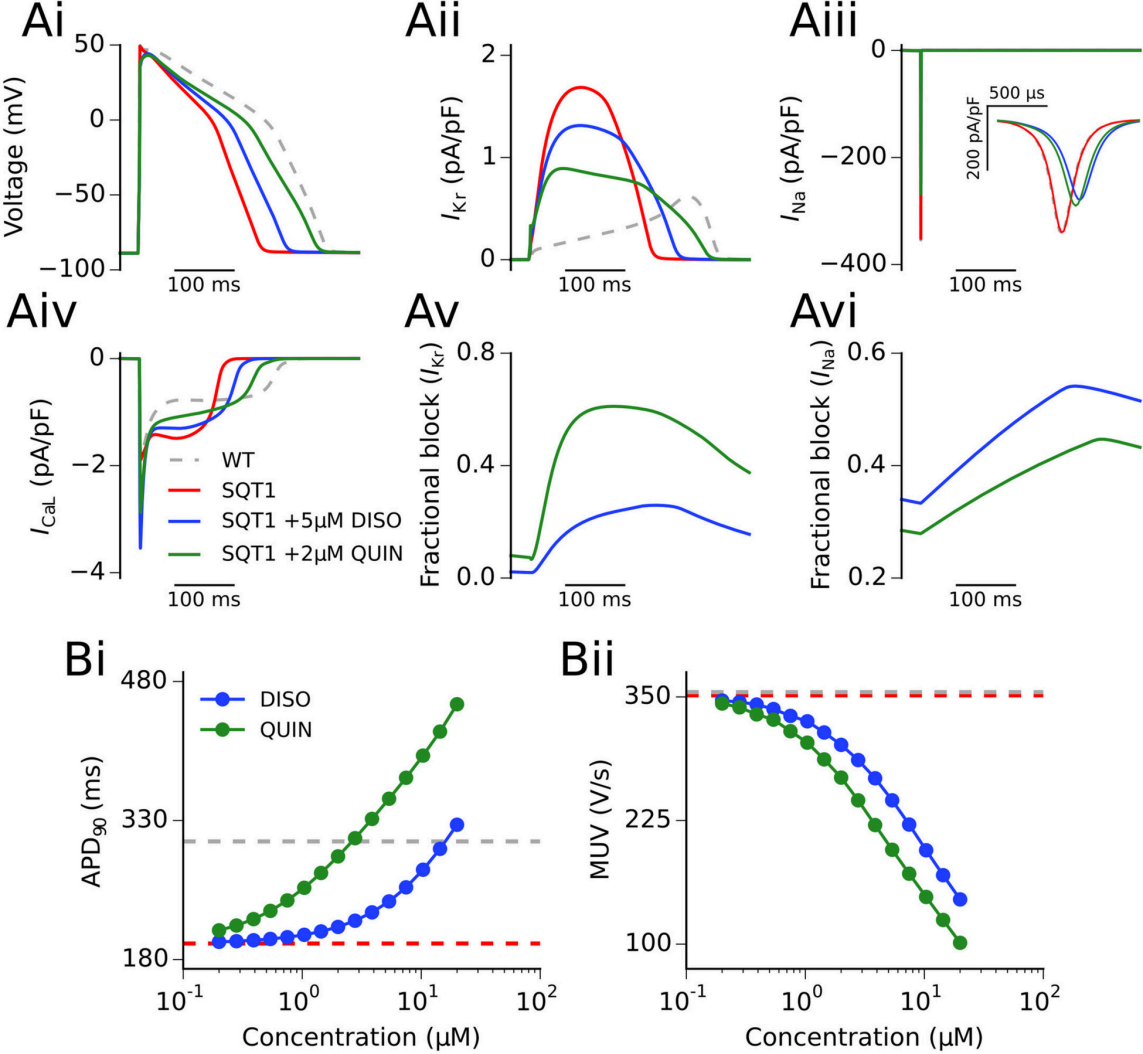
Single cell action potentials and current traces. **(Ai)** Endocardial action potential (AP) at 1 Hz under WT (silver, dashed line), SQT1 (red, solid line), SQT1 + 5μM disopyramide (DISO) (blue, solid line), and SQT1 + 2μM quinidine (QUIN) (green, solid line) conditions. Corresponding current traces are shown for *I*_Kr_ **(Aii)**, *I*_Na_ **(Aiii)**, and *I*_CaL_ **(Aiv)**. The degree of fractional block of *I*_Kr_ and *I*_Na_ is shown in **(Av,Avi)**, respectively. The concentration dependence of the single cell AP duration at 90% repolarization (APD_90_) and maximum upstroke velocity (MUV) is shown in **(Bi,Bii)**, respectively.

Figure 3A shows the actions of a representative concentration of disopyramide and quinidine (5 and 2 μM, respectively) on an endocardial ventricular cell AP and current profiles in the SQT1 condition at 1 Hz (see section Methods for consideration of concentrations used). It can be seen that both disopyramide and quinidine prolonged the action potential duration (APD) (Figure 3Ai; 238.3 and 289.5 ms upon application of 5 μM disopyramide and 2 μM quinidine, respectively, vs. 195.3 ms in the drug-free SQT1 condition) due to a considerable reduction in *I*_Kr_, which prolongs phase 3 repolarization (Figure 3Aii). Both drugs reduced the AP overshoot potential due to reduced *I*_Na_ (Figure 3Aiii), whereas only quinidine exhibited an effect on *I*_CaL_ at the concentrations shown (Figure 3Aiv). Figures 3Av,Avi show the fractional block of *I*_Kr_ and *I*_Na_, respectively, during the AP. The fractional block of *I*_Kr_ in the presence of quinidine was greater than that of disopyramide, even though the concentration was lower, as the IC_50_ for *I*_Kr_ block is roughly an order of magnitude lower (McPate et al., 2008). As both drugs block sodium channels with similar potencies (Koumi et al., 1992), 5 μM disopyramide produced a larger fractional block of *I*_Na_ than 2 μM quinidine.

Figure 3B shows the effects on the single cell APD and maximum upstroke velocity (MUV) for 15 logarithmically-spaced free concentrations of disopyramide and quinidine (ranging from 0.2 to 20 μM) in the SQT1 condition. It can be seen in Figure 3Bi that both drugs prolonged the APD in a dose-dependent manner, with quinidine prolonging the APD to a greater extent than disopyramide. Both drugs also reduced the single cell MUV in a dose-dependent manner (Figure 3Bii), with quinidine producing a slightly larger reduction in MUV across all concentrations investigated. Figure S4 shows the effect of disopyramide and quinidine on the restitution of the APD. Both drugs exhibited a degree of reverse frequency dependence (i.e., larger prolongation of the APD at longer cycle lengths) in the setting of SQT1, which was more prominent for quinidine over the range of concentrations tested.

### 1D transmural ventricular strand investigations

**Figure 4:**
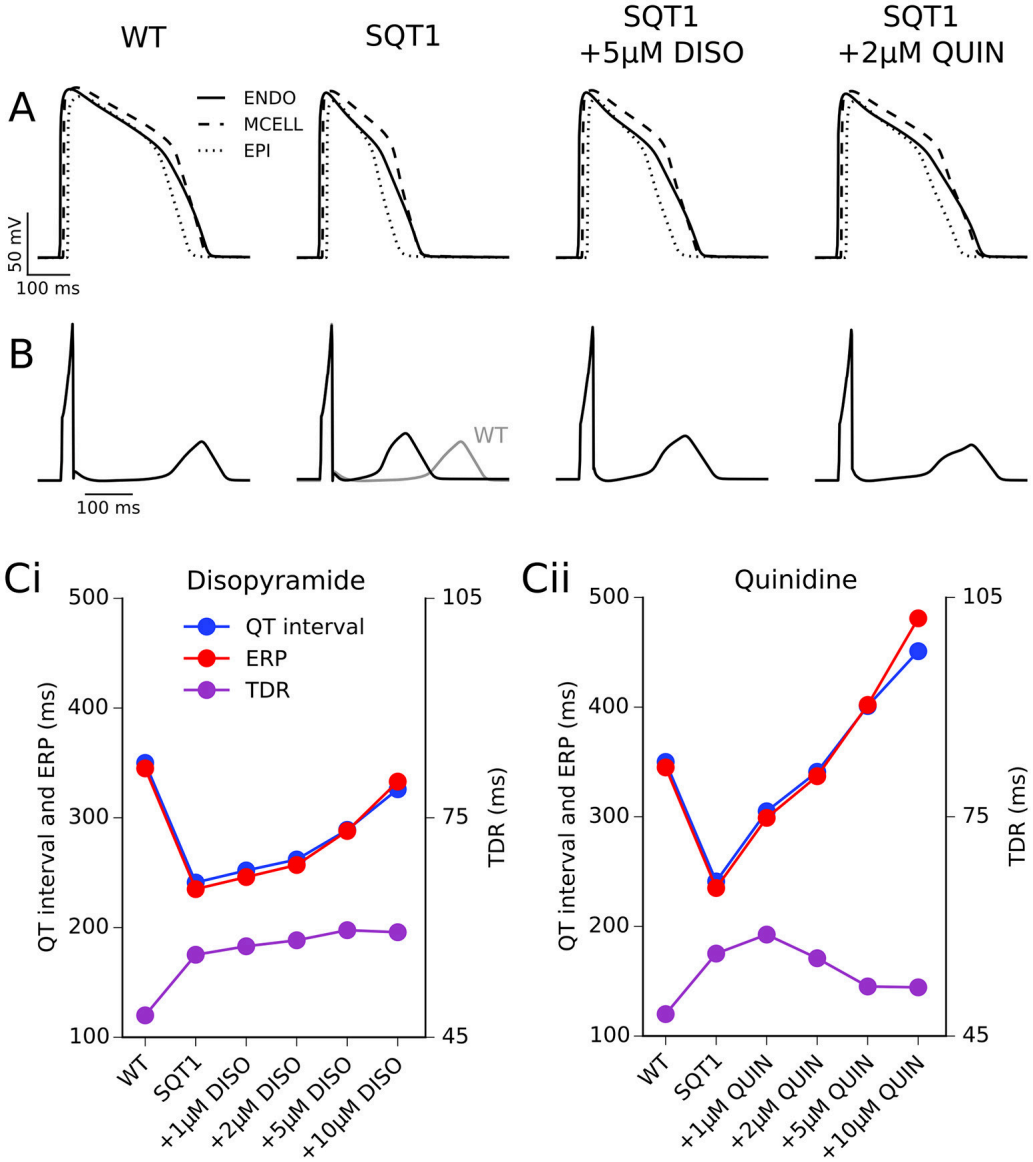
1D transmural ventricular strand simulations. **(A)** Endocardial (solid line), mid-myocardial (dashed line), and epicardial (dotted line) action potentials (APs) at 1 Hz under WT, SQT1, SQT1 + 5 μM disopyramide (DISO), and SQT1 + 2 μM quinidine (QUIN) conditions **(A)**. Corresponding pseudo ECGs (pECGs) are shown in **(B)**, with the WT pECG (shown in gray) superimposed under that of the SQT1 condition for comparison. The QT interval, effective refractory period (ERP), and maximal transmural dispersion of repolarization (TDR) for WT, SQT1, and SQT1 + varying concentrations of disopyramide (DISO) and quinidine (QUIN) is shown in **(Ci,Cii)**, respectively. Note that the *y* axis scale for QT interval and ERP is different to that of TDR.

The effects of 5 μM disopyramide and 2 μM quinidine on coupled cell APs in a 1D strand tissue model and the corresponding pECG waveforms can be seen in Figures 4A,B. As observed with the results on the single cell APD, 2 μM quinidine produced a more marked prolongation of the QT interval than did 5 μM disopyramide in the SQT1 condition (341 vs. 289 ms, compared to 241 ms in the drug-free SQT1 condition). Figure 4B shows the effects of four different concentrations of disopyramide and quinidine (1, 2, 5, 10 μM) on the QT interval, ERP, and transmural dispersion of repolarization (TDR) in the 1D strand at 1 Hz (the concentration range of 1–10 μM disopyramide/quinidine is shown on dose-response curves in Figures 1, 2 by light blue shaded regions). Both drugs caused a dose-dependent increase in the QT interval and ERP (Figures 4Ci,Cii). In agreement with the single cell results, prolongation of the QT interval and ERP was greater with quinidine than with disopyramide. The maximal TDR, which was higher in the SQT1 condition than WT, was not restored by disopyramide, whereas a modest reduction was observed for high concentrations of quinidine. Although, quinidine increased the ERP to a greater extent than disopyramide, it also produced a greater slowing of the conduction velocity (CV), which affects the wavelength (WL) of re-entry, given by WL = CV × ERP. Nonetheless, the WL was consistently prolonged to a greater extent with quinidine than with disopyramide. A summary of the effects of four different concentrations of disopyramide and quinidine on the QT interval, ERP, CV, and WL at 1 Hz is given in Table 2.

**Table 2:** A summary of effects of disopyramide and quinidine on tissue properties at 1 Hz.

		**QT (ms)**	**ERP (ms)**	**CV (cm/s)**	**WL (mm)**
**Drug-free**	**WT**	350	345	60.3	208.1
	**SQT1**	241	235	60.2	141.4
**SQT1** + **disopyramide**	**1** μ**M**	252	246	57.8	142.3
	**2** μ**M**	262	257	56.0	143.8
	**5** μ**M**	289	288	51.3	147.6
	**10** μ**M**	326	333	45.9	152.9
**SQT1** + **quinidine**	**1** μ**M**	305	299	56.2	168.2
	**2** μ**M**	341	337	52.9	178.4
	**5** μ**M**	401	402	46.4	186.5
	**10** μ**M**	451	481	40.3	194.0

*Comparison of QT interval, effective refractory period (ERP), conduction velocity (CV), and excitation wavelength (WL) in drug-free WT and SQT1 conditions, as well as upon application of 4 different concentrations of disopyramide and quinidine in the setting of SQT1*.

### Ionic contributions to drug actions of disopyramide and quinidine

**Figure 5:**
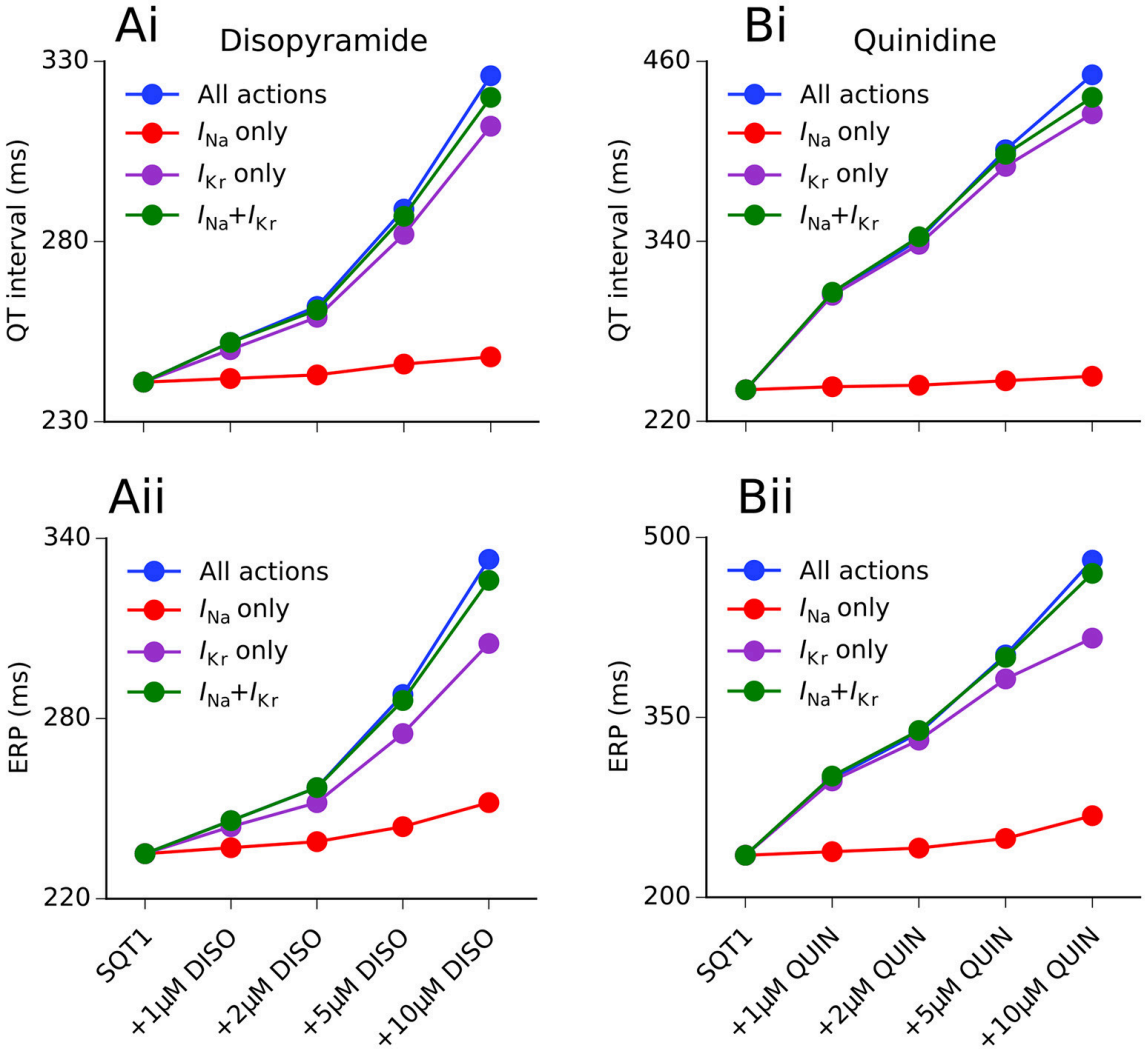
Individual ionic contributions of *I*_Kr_ and *I*_Na_ to QT interval and ERP prolongation by disopyramide and quinidine. The dose-dependent increase in the QT interval **(Ai,Bi)** and effective refractory period (ERP) **(Aii,Bii)** is shown for disopyramide (DISO) **(A)** and quinidine (QUIN) **(B)** for the following scenarios: all combined actions (blue), actions on *I*_Na_ only (red), actions on *I*_Kr_ only (purple), and combined actions on *I*_Na_ and *I*_Kr_ only (green). Simulations conducted at 1 Hz.

We hypothesized that the QT interval prolonging effects of disopyramide and quinidine in the setting of SQT1 were mainly due to *I*_Kr_ block, as a “gain-of-function” in *I*_Kr_ is responsible for the SQT1 phenotype. This would explain why quinidine prolongs the APD and QT interval to a greater extent than disopyramide, as it is a more potent inhibitor of the hERG channel (McPate et al., 2006, 2008). In order to investigate this, we computed the effects of both drugs on the QT interval and ERP for four different concentrations (1, 2, 5, 10 μM) with combined multi-channel actions, as well as three different hypothetical scenarios: (i) *I*_Kr_ block alone; (ii) *I*_Na_ block alone; and (iii) *I*_Kr_ + *I*_Na_ block only. A summary of these investigations is given in Figure 5.

Disopyramide block of *I*_Kr_ alone produced a relatively large increase in the QT interval and the ERP, whereas *I*_Na_ block alone produced only a modest increase in the ERP and very small increase in the QT interval (due to widening of the QRS complex). The combination of *I*_Kr_ and *I*_Na_ block produced a synergistic increase in the ERP, e.g., disopyramide block of *I*_Kr_ and *I*_Na_ alone (10 μM) prolonged the ERP by 29.8 and 7.2%, respectively, compared to 38.7% for combined *I*_Kr_ and *I*_Na_ block. The combined effects of 10 μM disopyramide on *I*_Kr_ and *I*_Na_ accounted for 92.9 and 92.8% of QT interval and ERP prolongation, respectively, compared with all multi-channel actions, suggesting that disopyramide block of *I*_to_ and *I*_Ks_ played only minor roles in prolonging the APD.

In the case of quinidine, *I*_Kr_ block alone accounted for 98.4% of the QT prolongation at a concentration of 1 μM, and 87.6% at 10 μM. The effects of *I*_Na_ block alone were comparatively minor, extending the QT interval by only 3.7% at a concentration of 10 μM. Prolongation of the ERP was slightly more dependent on *I*_Na_ block than QT prolongation, with the blocking actions of quinidine on *I*_Na_ accounting for 13.4% of total ERP prolongation at 10 μM. Unlike disopyramide, relative effects of combined block of *I*_Kr_ and *I*_Na_ by quinidine were dependent on the concentration. At low concentrations (1 and 2 μM), combined *I*_Na_ and *I*_Kr_ block produced greater prolongation of the QT interval and ERP than combined multi-channel actions, highlighting the role of L-type calcium and late sodium channel block in counterbalancing *I*_Kr_ block at these concentrations. At higher doses (5 and 10 μM), combined multi-channel actions produced a larger increase in the QT interval and ERP than combined block of *I*_Kr_ and *I*_Na_ alone, as quinidine block of *I*_Ks_ and *I*_K1_ also contributed to APD prolongation.

During simulated low-rate tachycardia (2 Hz), the contribution of *I*_Na_ block to total ERP prolongation at 10 μM compared to 1 Hz increased from 17.3 to 28.2% for disopyramide, and 13.4 to 25.3% for quinidine (see Figure S5), reflecting development of greater use dependent block of *I*_Na_ at faster rates.

### 3D left ventricular wedge simulations

In order to characterize drug effects on re-entry dynamics in the setting of SQT1, the effects of disopyramide and quinidine (concentrations of 1, 2, and 5 μM) on the DF and re-entrant excitation wave dynamics in the 3D ventricular wedge were quantified and compared to the drug-free SQT1 condition. In the absence of pharmacological modulation, initiated scroll waves were generally unstable and eventually degenerated into multiple, regenerative wavelets, characteristic of ventricular fibrillation (VF), as can be seen in representative Video S1. The average computed DF in the drug-free condition was 6.32 Hz, and re-entrant activity sustained for the full 5.0 s in all simulations (*n* = 6). Application of all concentrations of disopyramide tested reduced the DF in a dose-dependent manner, whereas likelihood of re-entry termination was not increased in a dose-dependent way, typically only occurring within the 5.0 s simulation period for concentrations of 1 and 2 μM (average DF and lifespan are summarized for all 3D simulations in Figure S6). An example of arrhythmia termination can be seen in representative Video S2, where addition of 1 μM disopyramide reduced the re-entry lifespan to ~1.4 s, and reduced the complexity of the electrical excitation wave pattern (i.e., reducing multiple wavelets to a single wave). At a concentration of 5 μM disopyramide using the same phase distribution initial conditions, the initiated scroll wave settled into a persistent single rotating scroll wave, characteristic of ventricular tachycardia (VT), as seen in Video S3. The initiated scroll wave also meandered to a much smaller extent, due to visibly slowed conduction through block of *I*_Na_. A summary of the averaged DF, lifespan of re-entry, and number of non-sustained arrhythmias (NST) following application of disopyramide and quinidine is given in Table 3.

**Table 3:** A summary of effects of disopyramide and quinidine on 3D wedge simulations.

	**DF (Hz)**	**Lifespan reentry (s)**		**DF (Hz)**	**Lifespan reentry (s)**
**SQT1**	6.32	5.00 (NST = 0)	**SQT1**	6.32	5.00 (NST = 0)
+**1** μ**M DISO**	5.99	4.00 (NST = 2)	+**1** μ**M QUIN**	5.03	4.97 (NST = 1)
+**2** μ**M DISO**	5.29	4.34 (NST = 2)	+**2** μ**M QUIN**	4.39	3.83 (NST = 3)
+**5** μ**M DISO**	4.26	5.00 (NST = 0)	+**5** μ**M QUIN**	3.15	4.93 (NST = 1)

*The averaged dominant frequency (DF) and lifespan of re-entrant excitation waves (NST denotes number of non-sustained arrhythmias) in drug-free SQT1 conditions is shown, as well as upon application of different concentrations of disopyramide (DISO) and quinidine (QUIN). In all cases n = 6*.

**Figure 6:**
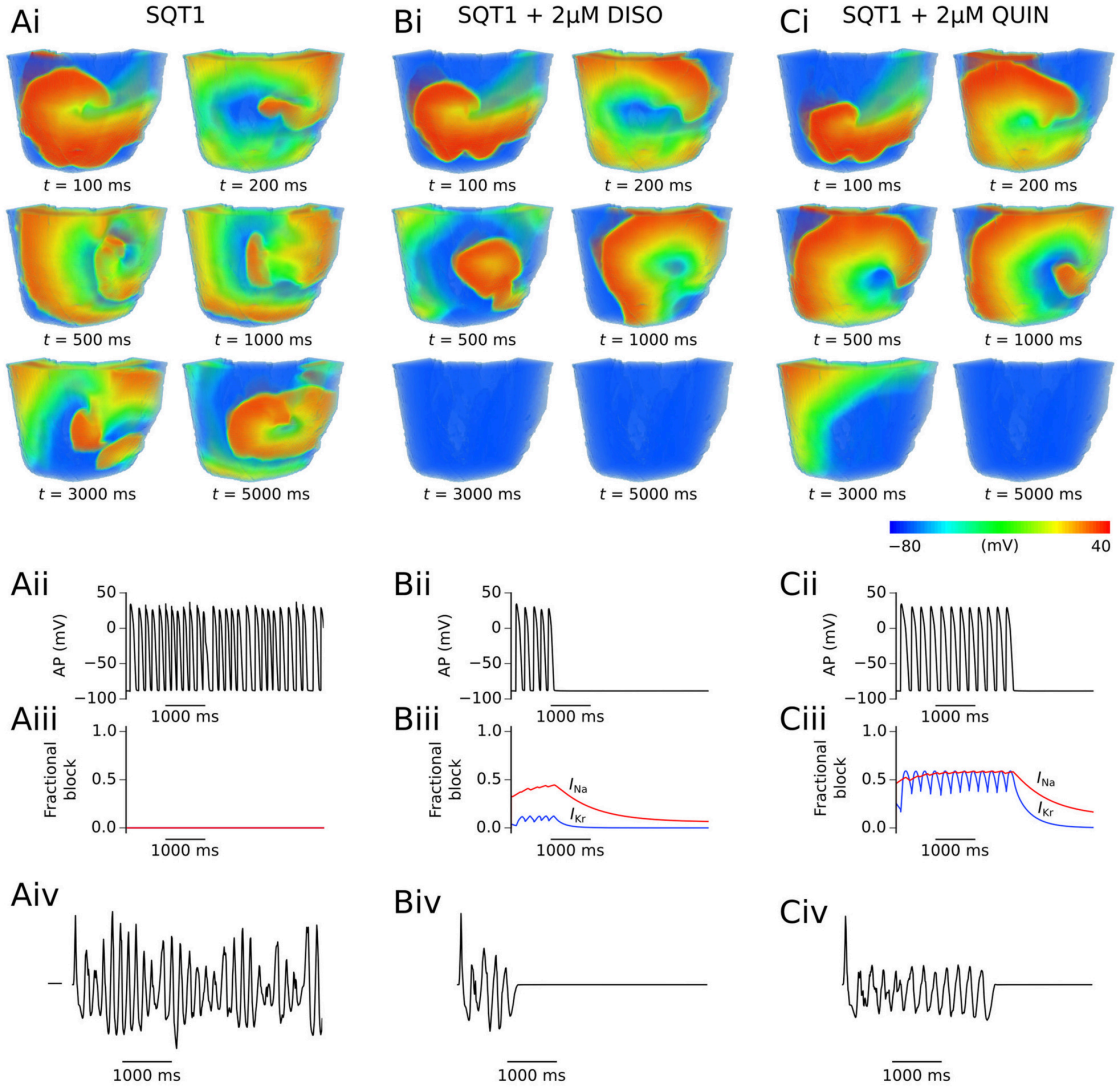
Representative snapshots of re-entry under SQT1 and pharmacological modulation conditions. The evolution of scroll waves following initiation of re-entry for time *t* = 100, 200, 500, 1,000, 3,000, and 5,000 ms is shown for SQT1 **(Ai)**, SQT1 + 2 μM disopyramide (DISO) **(Bi)**, and SQT1 + 2 μM quinidine (QUIN) **(Ci)** conditions from an epicardial aspect. In each case, localized AP excitations **(Aii,Bii,Cii)**, corresponding fractional block of *I*_Na_ (red) and *I*_Kr_ (blue) **(Aiii,Biii,Ciii)**, and pseudo ECG traces **(Aiv,Biv,Civ)** are shown.

Application of quinidine exerted a stronger inhibitory effect on mutant *I*_Kr_ in the setting of SQT1 than disopyramide at the same concentration, decreasing the DF to a larger extent. Similarly to disopyramide, it demonstrated the ability to terminate re-entrant excitations under certain conditions, as shown for 1 μM quinidine in representative Video S4, where the lifespan was reduced to ~4.8 s. Furthermore, it precluded the formation of a persistent VF-like electrical pattern, with initiated scroll waves typically settling into a slowly-rotating VT-like pattern at a higher concentration of 5 μM quinidine (e.g., see Video S5). Examples of arrhythmia termination by disopyramide and quinidine are given in Figure 6, which shows snapshots of re-entry for 2 μM disopyramide and quinidine alongside the drug-free SQT1 condition. In addition, localized AP traces extracted from the center of the 3D wedge, corresponding fractional block of *I*_Na_ and *I*_Kr_, and pECGs are shown. A summary of all pECGs from 3D wedge simulations is given in Figure S6, where it can be seen that in general the higher the concentration of disopyramide or quinidine, the more organized the waveform.

## Discussion

In this study we have characterized the effects of class Ia anti-arrhythmic agents disopyramide and quinidine in the setting of SQT1 using a hierarchy of virtual human ventricle models. This study builds on previous *in silico* work which has focused on QT interval shortening and arrhythmia substrates in the setting of SQT1 (Zhang and Hancox, 2004; Weiss et al., 2005; Adeniran et al., 2011), by using biophysically-detailed computational models to investigate the mode of action of two pharmacological agents which have demonstrated clinical effectiveness in partially reversing the SQT1 phenotype (Gaita et al., 2004; Wolpert et al., 2005; Schimpf et al., 2007; Giustetto et al., 2011).

### Main findings

**Figure 7:**
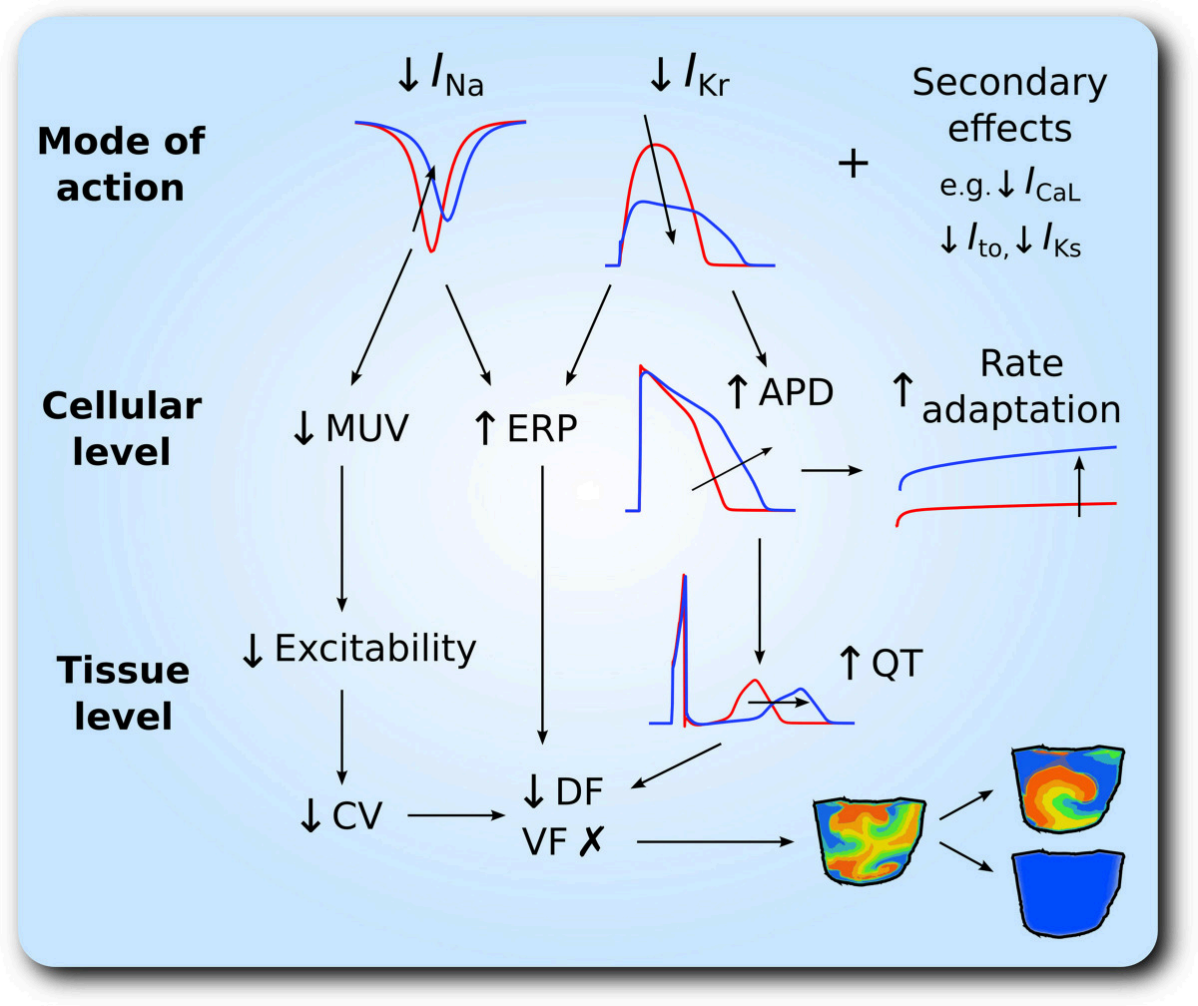
Schematic representation of anti-arrhythmic effects of disopyramide and quinidine in the setting of SQT1. The upper panel shows inhibition of *I*_Na_ and *I*_Kr_ as the principal mode of action of disopyramide and quinidine. At the cellular level, this corresponds to reduced maximum upstroke velocity (MUV), increased effective refractory period (ERP), action potential duration (APD), and rate adaptation, which is summarized in the middle panel. The lower panel shows consequent effects at the tissue level; reduced excitability and therefore reduced conduction velocity (CV), and prolonged QT interval, which translates to a reduction in the dominant frequency (DF) of re-entrant excitation waves and favors transition from multiple wavelet ventricular fibrillation (VF)- like wave patterns to single scroll wave re-entry and/or arrhythmia termination.

Our major findings are as follows. (1) In the setting of SQT1, both drugs caused a dose-dependent increase in the QT interval and ERP, and a dose-dependent decrease in the CV. Quinidine was more effective at restoring the QT interval to normal levels than disopyramide, due to more potent block of *I*_Kr_. (2) Although, disopyramide and quinidine exhibit multi-channel effects, only *I*_Kr_ and *I*_Na_ block were required to considerably prolong the QT interval and ERP. (3) Both drugs showed an anti-arrhythmic increase in the WL required to accommodate a re-entrant circuit. (4) Both drugs demonstrated a dose-dependent decrease in the DF of re-entry in 3D ventricular wedge simulations, which was greater for quinidine, whilst preventing the formation of chaotic, fibrillatory behavior, and occasionally terminating re-entrant waves. For patients who do not tolerate quinidine, disopyramide may offer an alternative pharmacological approach in SQT1. A schematic summary of the anti-arrhythmic effects of disopyramide and quinidine is given in Figure 7.

### Model validation

Hu et al. reported the average corrected QT (QT_c_) interval in probands with the N588K hERG mutation and affected relatives (*n* = 16) to be 284.7 ± 16.7 ms (Hu et al., 2017), which is significantly smaller than control QT_c_ intervals, e.g., 405.7 ± 30.2 ms (*n* = 149) given in Anttonen et al. (2009). Using these average QT_c_ intervals as a guide, this corresponds to a reduction of ~30% in N588K-mediated SQT1. In our 1D transmural human ventricle model, the SQT1 condition reduced the QT interval by ~31%, which agrees closely with this estimate from clinical observations. Moreover, the computed pECG accurately reproduced increased T wave amplitude in the setting of SQTS (Schimpf et al., 2005). The QT prolongation computed over the concentration range tested (1, 2, 5, 10 μM) was compared with a range of clinical measurements in Table S4, where good agreement is generally seen for concentrations of 1–5 μM.

### Anti-arrhythmic drug actions

At the single cell level, disopyramide and quinidine produced a dose-dependent increase in the APD_90_ and decrease in the MUV in the setting of SQT1. Both drugs also demonstrated some ability to restore rate adaptation, which is largely reduced in SQTS patients (Wolpert et al., 2005). In the multicellular 1D strand, a dose-dependent increase in the QT interval and decrease in the CV was observed, both of which were greater for quinidine. Reduced Na^+^ current which decreases CV also reduces cellular excitability and can thus suppress ventricular ectopic activity, which was one historic motivation for using class Ia anti-arrhythmic agents (Roden, 2014). Prolongation of the ERP and QT interval upon application of disopyramide and quinidine indicates that both agents were able to partially reverse the effects of SQT1, but not restore TDR to levels seen in the WT condition. In the case of quinidine, this finding is consistent with a previous study which showed inability of 10 μM quinidine to restore TDR in an experimental model of SQT1 (Patel and Antzelevitch, 2008).

By isolating individual and combined contributions of disopyramide and quinidine block of *I*_Na_ and *I*_Kr_ in the 1D tissue model, we demonstrated that only *I*_Kr_ block was necessary to considerably prolong the QT interval in the setting of SQT1 at a normal pacing rate. Prolongation of the ERP also relied heavily on *I*_Kr_ block, but increased synergistically when combined with *I*_Na_ block. The contribution of *I*_Na_ block to ERP prolongation increased further at a faster rate (2 Hz), due to greater development of use-dependent block of *I*_Na_. These findings suggest that combined multi-channel blocking effects of disopyramide or quinidine on other ionic currents such as *I*_CaL_, *I*_Ks_, *I*_to_, *I*_K1_, and *I*_NaL_ play only minor roles at therapeutic concentrations. Our study also substantiates the notion that *I*_Kr_ blockers which do not rely strongly on channel inactivation for binding should be considered desirable candidates for pharmacological treatment of N588K-mediated SQT1 (McPate et al., 2006, 2008; Perrin et al., 2008).

In 3D wedge simulations we investigated the effects of varying concentrations of disopyramide and quinidine on re-entry dynamics, with several initial condition (*n* = 6) scroll waves investigated (Figure S3). In the SQT1 condition, the single initiated scroll wave generally degenerated into multiple wavelets, consistent with clinical observations of VF in SQT1 patients (Brugada et al., 2004; Hu et al., 2017). Application of disopyramide and quinidine decreased the DF, and prevented formation of persistent VF-like electrical wave patterns; both effects which are anti-arrhythmic. A summary of all 3D simulations is given in Figure S6, where it can be seen from the pECGs that application of both drugs generally favored the transition from polymorphic VT/VF-like waveforms in the drug-free SQT1 condition, to more organized monomorphic VT-like waveforms, especially for higher concentrations. Although low concentrations of disopyramide and quinidine (1 and 2 μM) demonstrated some efficacy in terminating re-entrant excitation waves, paradoxically the higher concentration (5 μM) was less effective, as it caused greater slowing of the CV, which can facilitate the maintenance of arrhythmias (Nattel, 1998). However, it should be emphasized that we only investigated very short-term (5.0 s) re-entry dynamics. Furthermore, we investigated only the effects of pharmacological modulation on maintenance and not initiation of scroll waves. It is relevant in this regard that the Na^+^ blocking actions of both drugs reduce cellular excitability, meaning a stronger stimulus is required to initiate APs, and the observed increase in re-entry WL increases the spatial stimulus requirement to initiate re-entry (Adeniran et al., 2011).

### Potential pro-arrhythmic drug actions

The pro-arrhythmic potential of QT interval prolongation through hERG channel block has been known for some time (Nattel, 1998). Anti-arrhythmic agents may pose a risk even in the absence of disease such as ischemia or infarction (Nattel, 1998), which has resulted in a decline in the use of class Ia anti-arrhythmic drugs in recent years (Roden, 2014). In the absence of abbreviated repolarization, both disopyramide and quinidine have been associated with acquired long QT syndrome and the life-threatening ventricular arrhythmia *torsades de pointes* (Roden and Woosley, 1983). However, it has been suggested that the QT prolonging effect of these drugs, which is an unwanted side effect in treatment of conditions such as atrial arrhythmias, is desirable in the setting of SQT1 (Dumaine and Antzelevitch, 2006).

In supplemental investigations we found that both disopyramide and quinidine demonstrated reverse frequency dependence (i.e., larger APD prolongation at slower pacing frequencies), which had the effect of partially restoring rate adaptation which is reduced to a large extent in SQT1 (Wolpert et al., 2005; see Figure S4). In addition, using a slow pacing protocol (0.5 Hz) in mid-myocardial cells, we evaluated in the setting of SQT1 under variant parameter combinations the likelihood of development of EADs, which may be a mechanism for *torsades de pointes* (Weiss et al., 2010). We found that under normal conditions EADs were more readily inducible by 5 μM quinidine than disopyramide due to more potent block of *I*_Kr_. In the setting of SQT1, development of EADs by quinidine was much less likely than under WT conditions, occurring only in cases of significantly reduced repolarization reserve or *I*_CaL_ agonism. 5 μM of disopyramide did not induce EADs under any parameter combinations in SQT1 conditions. The results of these simulations, summarized in Figures S7, S8, suggest that neither drug poses a torsadogenic risk in the setting of SQT1 within the range of concentrations used in this study, especially disopyramide. Moreover, these simulations provide some indications about the concurrent use of other drugs with disopyramide and quinidine in SQT1. For example, our results suggest that use of disopyramide and quinidine in conjunction with additional hERG channel blockers or calcium channel agonists is contraindicated.

### Comparison with other simulation data

A recent study investigated effects of quinidine, disopyramide, and E-4031 on SQT1 in human ventricle computer models by implementing simple “pore block” theory (Luo et al., 2017). Whilst the models were able to predict the clinical effectiveness of quinidine in the setting of SQT1 (Wolpert et al., 2005), they did not reproduce favorable effects of disopyramide observed clinically (Schimpf et al., 2007; Giustetto et al., 2011). This is potentially due to differences in the models used—that study used the TP model (ten Tusscher and Panfilov, 2006) whereas the present study utilized the ORd model (O'Hara et al., 2011). In supplemental investigations, the degree of APD/QT interval prolongation at 1 Hz under application of disopyramide and quinidine in both “original” and “optimized” forms of the ORd and TP models (according to modifications detailed in Mann et al., 2016) was assessed. We found that “optimized” forms of the ORd and TP models showed convergent behavior (Figures S9, S10), whereas the original TP model underestimated the relative degree of disopyramide-induced APD/QT prolongation.

Another potential reason for the ineffectiveness of disopyramide in the Luo et al. study is due to lack of consideration of drug binding kinetics. In particular, the simple “pore block” approach used for *I*_Na_ in that study did not account for the use dependence of sodium channel block (Koumi et al., 1992), rendering it simplistic for arrhythmia simulations, and unable to recapitulate the increase in sodium channel block by class I drugs at fast rates (Roden, 2014). The importance of considering use-dependent block of *I*_Na_ on modulation of the ERP at fast racing rates is highlighted in Figure S11. The present study utilized drug binding kinetic models for *I*_Na_ and *I*_Kr_, which were shown to be the primary determinants of QT interval and ERP prolongation, and reproduced quantitatively QT prolongation observed with disopyramide in SQT1 (Schimpf et al., 2007; Giustetto et al., 2011) at clinically-relevant concentrations (see Table S4). Whilst the present study did not incorporate drug binding kinetics for all affected ion channel currents due to lack of experimental data, it nonetheless represents a significant advance over the previous simulation study (Luo et al., 2017).

### Limitations

There are a few limitations to consider in interpreting the results of this study. Firstly, *I*_Kr_/hERG block aside, most of the IC_50_ values from which the drug models were constructed were recorded from non-human, mammalian species due to lack of human experimental data. For the same reason, state-dependent drug binding models for *I*_Na_ and *I*_Kr_ only were considered, with simple pore blocks being used for other currents such as *I*_to_, *I*_Ks_, and *I*_CaL_. In addition, the structure of the drug-bound Markov model of *I*_Kr_ used (Perrin et al., 2008) did not include binding to closed states, the possibility of which cannot entirely be excluded, though both quindine and disopyramide are clearly predominantly gated state-dependent drugs that require access to the hERG channel pore to bind (Lees-Miller et al., 2000; El Harchi et al., 2012).

The heterozygous WT-N588K formulation used throughout the study is based on the simplifying assumption that SQT1 mutant *I*_Kr_ behaves in the same way as an equal mix of homomeric WT and mutant channels. In reality, the channel population may be more complex, with each channel comprising both WT and SQT1 mutant hERG channel subunits. Nevertheless, our “SQT1” formulation reproduced QT interval shortening to an extent that was in agreement with clinical measurements, as well as increased T wave amplitude which is commonly observed in SQTS patients (Anttonen et al., 2009), thereby supporting the approach adopted here.

Finally, some care must be exercised in interpreting the results from the 3D ventricle wedge geometry which, despite offering advantages over previously-used simplified geometries (Luo et al., 2017), excludes realistic boundaries and lacks features such as a Purkinje fiber network which may play a role in arrhythmogenesis.

## Conclusions

This study used computational modeling to dissect ionic mechanisms underlying QT prolongation and anti-arrhythmic actions of disopyramide and quinidine on SQT1 in human ventricles. Both drugs were shown to be effective inhibitors of mutant hERG channels, and demonstrated efficacy in partially reversing the SQT1 phenotype. Furthermore, both disopyramide and quinidine exhibited anti-arrhythmic effects in the 3D left ventricular wedge. These, along with our EAD simulations, substantiate the notion that drugs which can be life-*threatening* in the context of normal repolarization can be life-*saving* in the context of abbreviated repolarization (Dumaine and Antzelevitch, 2006). This study further establishes disopyramide as a potential suitable alternative for SQT1 patients who do not tolerate quinidine well, and provides new insights into class Ia-mediated pharmacological treatments in SQT1.

## Author contributions

DW, JH, and HZ conceived the experiments. DW developed and validated computer models. DW performed numerical experiments and analysis. HN and AB contributed computing resources. All authors wrote the manuscript.

### Conflict of interest statement

The authors declare that the research was conducted in the absence of any commercial or financial relationships that could be construed as a potential conflict of interest.

## References

[B1] Adeniran I. McPate M. J. Witchel H. J. Hancox J. C. Zhang H. (2011). Increased vulnerability of human ventricle to re-entrant excitation in hERG-linked variant 1 short QT syndrome. *PLoS Comput*. Biol. 7:e1002313 10.1371/journal.pcbi.1002313 PMC3240585 22194679

[B2] Adeniran I. Whittaker D. G. Harchi A. E. Hancox J. C. Zhang H. (2017). *In silico* investigation of a KCNQ1 mutation associated with short QT syndrome. Sci. Rep. 7:8469. 10.1038/s41598-017-08367-2 28814790 PMC5559555

[B3] Anttonen O. Junttila J. Giustetto C. Gaita F. Linna E. Karsikas M.. (2009). T-wave morphology in short QT syndrome. Ann. Noninvasive Electrocardiol. 14, 262–267. 10.1111/j.1542-474X.2009.00308.x 19614638 PMC6932637

[B4] Benson A. P. Bernus O. Dierckx H. Gilbert S. H. Greenwood J. P. Holden A. V.. (2011). Construction and validation of anisotropic and orthotropic ventricular geometries for quantitative predictive cardiac electrophysiology. Interface Focus 1, 101–116. 10.1098/rsfs.2010.0005 22419977 PMC3262240

[B5] Biktashev V. N. Holden A. V. (1998). Reentrant waves and their elimination in a model of mammalian ventricular tissue. Chaos Interdiscip. J. Nonlinear Sci. 8, 48–56. 10.1063/1.166307 12779709

[B6] Brennan T. Fink M. Rodriguez B. (2009). Multiscale modelling of drug-induced effects on cardiac electrophysiological activity. Eur. J. Pharm. Sci. 36, 62–77. 10.1016/j.ejps.2008.09.013 19061955

[B7] Brugada R. Hong K. Dumaine R. Cordeiro J. Gaita F. Borggrefe M.. (2004). Sudden death associated with short-QT syndrome linked to mutations in HERG. Circulation 109, 30–35. 10.1161/01.CIR.0000109482.92774.3A 14676148

[B8] Clayton R. H. Bernus O. Cherry E. M. Dierckx H. Fenton F. H. Mirabella L.. (2011). Models of cardiac tissue electrophysiology: progress, challenges and open questions. Prog. Biophys. Mol. Biol. 104, 22–48. 10.1016/j.pbiomolbio.2010.05.008 20553746

[B9] Colman M. A. Ni H. Liang B. Schmitt N. Zhang H. (2017). *In silico* assessment of genetic variation in KCNA5 reveals multiple mechanisms of human atrial arrhythmogenesis. PLOS Comput. Biol. 13:e1005587. 10.1371/journal.pcbi.1005587 28622331 PMC5493429

[B10] Dumaine R. Antzelevitch C. (2006). Disopyramide: although potentially life-threatening in the setting of long QT, could it be life-saving in short QT syndrome? J. Mol. Cell. Cardiol. 41, 421–423. 10.1016/j.yjmcc.2006.06.070 16863649 PMC1989772

[B11] El Harchi A. Zhang Y. H. Hussein L. Dempsey C. E. Hancox J. C. (2012). Molecular determinants of hERG potassium channel inhibition by disopyramide. J. Mol. Cell. Cardiol. 52, 185–195. 10.1016/j.yjmcc.2011.09.021 21989164

[B12] Gaita F. Giustetto C. Bianchi F. Schimpf R. Haissaguerre M. Calò L.. (2004). Short QT syndrome: pharmacological treatment. J. Am. Coll. Cardiol. 43, 1494–1499. 10.1016/j.jacc.2004.02.034 15093889

[B13] Gima K. Rudy Y. (2002). Ionic current basis of electrocardiographic waveforms a model study. Circ. Res. 90, 889–896. 10.1161/01.RES.0000016960.61087.86 11988490 PMC1847799

[B14] Giustetto C. Monte F. D. Wolpert C. Borggrefe M. Schimpf R. Sbragia P.. (2006). Short QT syndrome: clinical findings and diagnostic–therapeutic implications. Eur. Heart J. 27, 2440–2447. 10.1093/eurheartj/ehl185 16926178

[B15] Giustetto C. Schimpf R. Mazzanti A. Scrocco C. Maury P. Anttonen O.. (2011). Long-term follow-up of patients with short QT syndrome. J. Am. Coll. Cardiol. 58, 587–595. 10.1016/j.jacc.2011.03.038 21798421

[B16] Giustetto C. Scrocco C. Giachino D. Rapezzi C. Mognetti B. Gaita F. (2015). The lack of effect of sotalol in short QT syndrome patients carrying the T618I mutation in the KCNH2 gene. Hear. Case Rep. 1, 373–378. 10.1016/j.hrcr.2015.07.001 28491588 PMC5419677

[B17] Hanada E. Ohtani H. Hirota M. Uemura N. Nakaya H. Kotaki H.. (2003). Inhibitory effect of erythromycin on potassium currents in rat ventricular myocytes in comparison with disopyramide. J. Pharm. Pharmacol. 55, 995–1002. 10.1211/0022357021459 12906757

[B18] Harrell D. T. Ashihara T. Ishikawa T. Tominaga I. Mazzanti A. Takahashi K.. (2015). Genotype-dependent differences in age of manifestation and arrhythmia complications in short QT syndrome. Int. J. Cardiol. 190, 393–402. 10.1016/j.ijcard.2015.04.090 25974115

[B19] Hu D. Li Y. Zhang J. Pfeiffer R. Gollob M. H. Healey J. (2017). The phenotypic spectrum of a mutation hotspot responsible for the short QT syndrome. JACC Clin. Electrophysiol. 3, 727–743. 10.1016/j.jacep.2016.11.013 29759541

[B20] Kang J. Chen X.-L. Wang L. Rampe D. (2001). Interactions of the antimalarial drug mefloquine with the human cardiac potassium channels KvLQT1/minK and HERG. J. Pharmacol. Exp. Ther. 299, 290–296. 11561091

[B21] Koumi S. Sato R. Katori R. Hisatome I. Nagasawa K. Hayakawa H. (1992). Sodium channel states control binding and unbinding behaviour of antiarrhythmic drugs in cardiac myocytes from the guinea pig. Cardiovasc. Res. 26, 1199–1205. 10.1093/cvr/26.12.1199 1337728

[B22] Kramer J. Obejero-Paz C. A. Myatt G. Kuryshev Y. A. Bruening-Wright A. Verducci J. S.. (2013). MICE models: superior to the HERG model in predicting torsade de pointes. Sci. Rep. 3:2100. 10.1038/srep02100 23812503 PMC3696896

[B23] Lees-Miller J. P. Duan Y. Teng G. Q. Duff H. J. (2000). Molecular determinant of high-affinity dofetilide binding to HERG1 expressed in xenopus oocytes: involvement of S6 sites. Mol. Pharmacol. 57, 367–374. 10648647

[B24] Loewe A. Wilhelms M. Fischer F. Scholz E. P. Dössel O. Seemann G. (2014). Arrhythmic potency of human ether-à-go-go-related gene mutations L532P and N588K in a computational model of human atrial myocytes. Europace 16, 435–443. 10.1093/europace/eut375 24569898

[B25] Luo C. H. Rudy Y. (1994). A dynamic model of the cardiac ventricular action potential. I. Simulations of ionic currents and concentration changes. Circ. Res. 74, 1071–1096. 10.1161/01.RES.74.6.1071 7514509

[B26] Luo C. Wang K. Zhang H. (2017). *In silico* assessment of the effects of quinidine, disopyramide and E-4031 on short QT syndrome variant 1 in the human ventricles. PLoS ONE 12:e0179515. 10.1371/journal.pone.0179515 28632743 PMC5478111

[B27] Mann S. A. Imtiaz M. Winbo A. Rydberg A. Perry M. D. Couderc J.-P.. (2016). Convergence of models of human ventricular myocyte electrophysiology after global optimization to recapitulate clinical long QT phenotypes. J. Mol. Cell. Cardiol. 100, 25–34. 10.1016/j.yjmcc.2016.09.011 27663173

[B28] McPate M. J. Duncan R. S. Hancox J. C. Witchel H. J. (2008). Pharmacology of the short QT syndrome N588K-hERG K^+^ channel mutation: differential impact on selected class I and class III antiarrhythmic drugs. Br. J. Pharmacol. 155, 957–966. 10.1038/bjp.2008.325 18724381 PMC2597231

[B29] McPate M. J. Duncan R. S. Milnes J. T. Witchel H. J. Hancox J. C. (2005). The N588K-HERG K^+^ channel mutation in the “short QT syndrome”: mechanism of gain-in-function determined at 37°C. Biochem. Biophys. Res. Commun. 334, 441–449. 10.1016/j.bbrc.2005.06.112 16011830

[B30] McPate M. J. Duncan R. S. Witchel H. J. Hancox J. C. (2006). Disopyramide is an effective inhibitor of mutant HERG K^+^ channels involved in variant 1 short QT syndrome. J. Mol. Cell. Cardiol. 41, 563–566. 10.1016/j.yjmcc.2006.05.021 16842817

[B31] McPate M. J. Zhang H. Adeniran I. Cordeiro J. M. Witchel H. J. Hancox J. C. (2009). Comparative effects of the short QT N588K mutation at 37 degrees C on hERG K^+^ channel current during ventricular, Purkinje fibre and atrial action potentials: an action potential clamp study. J. Physiol. Pharmacol. 60, 23–41. 19439805

[B32] Mizobuchi M. Enjoji Y. Yamamoto R. Ono T. Funatsu A. Kambayashi D.. (2008). Nifekalant and disopyramide in a patient with short QT syndrome: evaluation of pharmacological effects and electrophysiological properties. Pacing Clin. Electrophysiol. 31, 1229–1232. 10.1111/j.1540-8159.2008.01169.x 18834480

[B33] Moreno J. D. Lewis T. J. Clancy C. E. (2016). Parameterization for *in-silico* modeling of ion channel interactions with drugs. PLoS ONE 11:e0150761. 10.1371/journal.pone.0150761 26963710 PMC4786197

[B34] Nattel S. (1998). Experimental evidence for proarrhythmic mechanisms of antiarrhythmic drugs. Cardiovasc. Res. 37, 567–577. 10.1016/S0008-6363(97)00293-9 9659440

[B35] Nenov N. I. Crumb W. J. Pigott J. D. Harrison L. H. Clarkson C. W. (1998). Quinidine interactions with human atrial potassium channels. Circ. Res. 83, 1224–1231. 10.1161/01.RES.83.12.1224 9851939

[B36] O'Hara T. Virág L. Varró A. Rudy Y. (2011). Simulation of the undiseased human cardiac ventricular action potential: model formulation and experimental validation. PLoS Comput. Biol. 7:e1002061. 10.1371/journal.pcbi.1002061 21637795 PMC3102752

[B37] Patel C. Antzelevitch C. (2008). Cellular basis for arrhythmogenesis in an experimental model of the SQT1 form of the short QT syndrome. Heart Rhythm 5, 585–590. 10.1016/j.hrthm.2008.01.022 18362027 PMC2361425

[B38] Paul A. A. Witchel H. J. Hancox J. C. (2001). Inhibition of HERG potassium channel current by the class 1a antiarrhythmic agent disopyramide. Biochem. Biophys. Res. Commun. 280, 1243–1250. 10.1006/bbrc.2001.4269 11162661

[B39] Paul A. A. Witchel H. J. Hancox J. C. (2002). Inhibition of the current of heterologously expressed HERG potassium channels by flecainide and comparison with quinidine, propafenone and lignocaine. Br. J. Pharmacol. 136, 717–729. 10.1038/sj.bjp.0704784 12086981 PMC1573407

[B40] Perrin M. J. Kuchel P. W. Campbell T. J. Vandenberg J. I. (2008). Drug binding to the inactivated state is necessary but not sufficient for high-affinity binding to human ether-à-go-go-related gene channels. Mol. Pharmacol. 74, 1443–1452. 10.1124/mol.108.049056 18701618

[B41] Plonsey R. Barr R. C. (2013). Bioelectricity: A Quantitative Approach. Springer Science & Business Media.

[B42] Roden D. M. (2014). Pharmacology and toxicology of Nav1.5-Class 1 anti-arrhythmic drugs. Card. Electrophysiol. Clin. 6, 695–704. 10.1016/j.ccep.2014.07.003 25395995 PMC4226533

[B43] Roden D. M. Woosley R. L. (1983). Class I antiarrhythmic agents: quinidine, procainamide and N-acetylprocainamide, disopyramide. Pharmacol. Ther. 23, 179–191. 10.1016/0163-7258(83)90012-8 6199801

[B44] Sagawa K. Mohri K. Shimada S. Shimizu M. Muramatsu J. (1997). Disopyramide concentrations in human plasma and saliva: comparison of disopyramide concentrations in saliva and plasma unbound concentrations. Eur. J. Clin. Pharmacol. 52, 65–69. 10.1007/s002280050250 9143870

[B45] Satoh H. (2000). Comparative actions of cibenzoline and disopyramide on I_Kr_ and I_Ks_ currents in rat sino-atrial nodal cells. Eur. J. Pharmacol. 407, 123–129. 10.1016/S0014-2999(00)00734-2 11050299

[B46] Schimpf R. Veltmann C. Giustetto C. Gaita F. Borggrefe M. Wolpert C. (2007). *In vivo* effects of mutant HERG K^+^ channel inhibition by disopyramide in patients with a short QT-1 syndrome: a pilot study. J. Cardiovasc. Electrophysiol. 18, 1157–1160. 10.1111/j.1540-8167.2007.00925.x 17711440

[B47] Schimpf R. Wolpert C. Gaita F. Giustetto C. Borggrefe M. (2005). Short QT syndrome. Cardiovasc. Res. 67, 357–366. 10.1016/j.cardiores.2005.03.026 15890322

[B48] Starmer C. F. Grant A. O. Strauss H. C. (1984). Mechanisms of use-dependent block of sodium channels in excitable membranes by local anesthetics. Biophys. J. 46, 15–27. 10.1016/S0006-3495(84)83994-6 6331543 PMC1434933

[B49] Szabó G. Szentandrássy N. Bíró T. Tóth B. I. Czifra G. Magyar J.. (2005). Asymmetrical distribution of ion channels in canine and human left-ventricular wall: epicardium versus midmyocardium. Pflüg. Arch. 450, 307–316. 10.1007/s00424-005-1445-z 15952036

[B50] ten Tusscher K. H. W. J. Panfilov A. V. (2006). Alternans and spiral breakup in a human ventricular tissue model. Am. J. Physiol. Heart Circ. Physiol. 291, H1088–H1100. 10.1152/ajpheart.00109.2006 16565318

[B51] Villafañe J. Atallah J. Gollob M. H. Maury P. Wolpert C. Gebauer R.. (2013). Long-term follow-up of a pediatric cohort with short QT syndrome. J. Am. Coll. Cardiol. 61, 1183–1191. 10.1016/j.jacc.2012.12.025 23375927

[B52] Weiss D. L. Seemann G. Sachse F. B. Dössel O. (2005). Modelling of short QT syndrome in a heterogeneous model of the human ventricular wall. Europace 7, S105–S117. 10.1016/j.eupc.2005.04.008 16102508

[B53] Weiss J. N. Garfinkel A. Karagueuzian H. S. Chen P.-S. Qu Z. (2010). Early afterdepolarizations and cardiac arrhythmias. Heart Rhythm 7, 1891–1899. 10.1016/j.hrthm.2010.09.017 20868774 PMC3005298

[B54] Whittaker D. G. Ni H. Harchi A. E. Hancox J. C. Zhang H. (2017). Atrial arrhythmogenicity of KCNJ2 mutations in short QT syndrome: insights from virtual human atria. PLOS Comput. Biol. 13:e1005593. 10.1371/journal.pcbi.1005593 28609477 PMC5487071

[B55] Wolpert C. Schimpf R. Giustetto C. Antzelevitch C. Cordeiro J. Dumaine R.. (2005). Further insights into the effect of quinidine in short QT syndrome caused by a mutation in HERG. J. Cardiovasc. Electrophysiol. 16, 54–58. 10.1046/j.1540-8167.2005.04470.x 15673388 PMC1474841

[B56] Wu L. Guo D. Li H. Hackett J. Yan G.-X. Jiao Z.. (2008). Role of late sodium current in modulating the proarrhythmic and antiarrhythmic effects of quinidine. Heart Rhythm 5, 1726–1734. 10.1016/j.hrthm.2008.09.008 19084812 PMC2669543

[B57] Zhang H. Hancox J. C. (2004). *In silico* study of action potential and QT interval shortening due to loss of inactivation of the cardiac rapid delayed rectifier potassium current. Biochem. Biophys. Res. Commun. 322, 693–699. 10.1016/j.bbrc.2004.07.176 15325285

[B58] Zhang Y. Hancox J. (2002). Mode-dependent inhibition by quinidine of Na^+^–Ca2^+^ exchanger current from guinea-pig isolated ventricular myocytes. Clin. Exp. Pharmacol. Physiol. 29, 777–781. 10.1046/j.1440-1681.2002.03731.x 12165041

