# Supplementary material for: Computational Analysis of the Mode of Action of Disopyramide and Quinidine on hERG-Linked Short QT Syndrome in Human Ventricles
Source: Front Physiol. 2017 Oct 4;8:759. doi: 10.3389/fphys.2017.00759 (PMC5649182; doi:10.3389/fphys.2017.00759)
Supplement: Supplementary file 6 [file DataSheet1.DOCX]

Supplementary Material

Computational analysis of the mode of action of disopyramide and quinidine on hERG-linked short QT syndrome in human ventricles

Dominic G. Whittaker, Haibo Ni, Alan P. Benson, Jules C. Hancox*, Henggui Zhang*

*** Correspondence:** Corresponding Author(s):

jules.hancox@bristol.ac.uk (JH); henggui.zhang@manchester.ac.uk (HZ)

# Supplementary Methods

## Updated drug-free Markov chain model of WT and SQT1 mutant *I*_Kr_

Our previous study utilised a Markov chain (MC) formulation of *I*_Kr_ to describe kinetics in wild type (WT) and SQT1 mutant conditions (Adeniran et al., 2011) at 37°C. In that study, the Broyden-Fletcher-Goldfarb-Shanno (BFGS) algorithm was used to minimise the least-squared difference between experimental and simulated *I*-*V* relations (McPate et al., 2005). In the present study, ‘multi-objective fitting’ using a bounded Nelder-Mead simplex optimisation routine which employed the Parallel Computing Toolbox in Matlab R2014a (Math Works, Inc.) was performed (optimisation code can be downloaded from (Moreno et al., 2016)), in order to further improve the kinetics of the MC model of *I*_Kr_ by simultaneously using multiple experimental protocols. Briefly, experimental data regarding the *I*-*V* relation, steady state activation and inactivation, and fully-activated *I*-*V* relation were gathered (McPate et al., 2005) and the same protocols used to generate simulated currents. The optimisation algorithm then compared the sum of the least-squared difference between experimental and simulated currents for each protocol, which was defined as the cost function. During each iteration the set of parameters describing the MC model was perturbed. When a parameter set yielding an acceptably low cost function was obtained (i.e. the error between the model prediction and experimental data fell below a predefined value), the routine terminated (Moreno et al., 2016). Updated model fits to the experimental data are shown in Figure S1, and kinetic parameters for the MC are given in Supplementary Table S1.

## Parameter estimation of drug binding rates to *I*_Kr_ and *I*_Na_

Parameter estimation of rate constants for drug binding of disopyramide and quinidine to *I*_Kr_ and *I*_Na_ was performed using the procedure outlined above (Moreno et al., 2016). In the case of drug binding to *I*_Kr_/hERG, binding and unbinding rates to activated and inactivated channel states, *k*_A_, *k*_I_, *l*_A_, and *l*_I_ were allowed to vary freely in order to recapitulate experimental data on dose-dependent steady state block (WT and SQT1) and mean fractional block during a pulse protocol (shown in manuscript Figure 1), and voltage-dependent block of hERG tail currents for disopyramide and quinidine at 37°C (McPate et al., 2008; Paul et al., 2001, 2002). Parameters are given in Supplementary Table S2.

For *I*_Na_, disopyramide and quinidine were assumed to bind to activated, inactivated, and resting states of the Luo-Rudy model of *I*_Na_ (Luo and Rudy, 1994), and ‘multi-objective fitting’ was performed based on the experimental data of (Koumi et al., 1992). Binding and unbinding parameters *k*_A_, *k*_I_, *k*_R_, *l*_A_, *l*_I_, and *l*_R_ were included in optimisation, and constrained based on the dose-dependent tonic and use dependent block (shown in manuscript Figure 2), the onset of use-dependent block, and the time course of unbinding from use dependent block (Koumi et al., 1992). Parameters are given in Supplementary Table S3.

## Human ventricular cell action potential models

In this study, the O’Hara-Rudy dynamic (ORd) human ventricle cell model was used for simulations (O’Hara et al., 2011). The native equations for *I*_Kr_ were replaced with that of our newly-developed MC formulation, with the channel conductance, *g*_Kr_, set to $1.1\cdot0.0135\cdot\left[ K^{+} \right]_{o}^{0.59}$, where $\left[ K^{+} \right]_{o}$ is the extracellular potassium concentration (Adeniran et al., 2011), in order to produce an action potential (AP) duration (APD) close to that of the original model. The equations for *I*_Na_ in the ORd model, which have been shown previously to yield unphysiologically-slow conduction velocities in tissue (Elshrif and Cherry, 2014), were replaced with that of the Luo-Rudy model (Luo and Rudy, 1994), with maximal channel conductance, *g*_Na_, set to 10.4 nS/pF in order to produce appropriate values of AP amplitude and upstroke velocity for human ventricular myocytes (Elshrif and Cherry, 2014).

Modifications to the ORd model described in (Mann et al., 2016) which were calibrated on the basis of clinical QT interval data in the long QT syndrome were applied to the ORd model, which gave a QT interval shortening and increased T wave amplitude in the N588K-mediated SQT1 condition consistent with clinical observations (Schimpf et al., 2005). Briefly, conductance scaling factors for the slow delayed rectifier potassium current (*G*_Ks_ = 5.75), L-type calcium current (*G*_CaL_ = 2.01), sodium-calcium exchanger (*G*_NCX_ = 2.95), and sodium-potassium pump (*G*_NaK_ = 9.12) were introduced in order to alter the balance of ionic currents responsible for the AP. The resulting updated ORd model gave an epicardial APD of ~253 ms at a cycle length of 1000 ms, close to that measured experimentally in human ventricular myocytes (Li et al., 1998).

Comparative simulations were performed with the ten Tusscher-Panfilov (TP) model of human ventricular myocytes (Tusscher and Panfilov, 2006), in both its ‘original’ and ‘optimised’ form (Mann et al., 2016). In the optimised form of the model, conductance scaling factors for the slow delayed rectifier potassium current (*G*_Ks_ = 0.41), rapid delayed rectifier potassium current (*G*_Kr_ = 2.65), L-type calcium current (*G*_CaL_ = 0.79), sodium-calcium exchanger (*G*_NCX_ = 2.08), sodium-potassium pump (*G*_NaK_ = 2.67), and late sodium current (*G*_NaL_ = 3.18) were introduced in order to alter the balance of ionic currents responsible for the AP, as described in (Mann et al., 2016).

## Multicellular tissue models

In multicellular tissue simulations, the initiation and propagation of APs was modelled using a monodomain approach (Clayton et al., 2011):

$$\frac{\partial V}{\partial t}=\nabla\left( \text{D}\nabla V \right)-\frac{\left( I_{\text{ion}}+I_{\text{stim}} \right)}{C_{\text{m}}}$$

where *V* is the transmembrane voltage, **D** is the global conductivity tensor (diffusion coefficient) describing the tissue conductivity, *I*_ion_ and *I*_stim_ are the total ionic current and applied stimulus current, respectively, and *C*_m_ is the membrane capacitance.

For one-dimensional (1D) simulations a strand of 100 nodes with spatial step Δ*x* = 0.15 mm and no-flux boundary conditions was used as described previously (Adeniran et al., 2011, 2017), with each node representing a 150-μm cylindrical cell. This gave a total length of 15 mm, which agrees well with human transmural ventricle widths (Drouin et al., 1995; Yan et al., 1998). The transmural strand composition was also identical to that described previously (Adeniran et al., 2011). The diffusion coefficient, **D**, which describes the spread of the membrane potential in reaction-diffusion systems, was set to 0.1171 mm^2^ms^-1^ as derived for human ventricles (Bueno-Orovio et al., 2008). The value of **D** was homogenous except for a five-fold decrease at the epicardial:mid-myocardial cell border (Adeniran et al., 2011; Gima and Rudy, 2002).

A supra-threshold stimulus was applied in the endocardial cell region in order to initiate an excitation wave in the 1D transmural strand. Conduction velocity was computed from nodes one-quarter and three-quarters of the way across the strand as the quotient of distance travelled and activation time difference (defined as point of maximum upstroke velocity). The effective refractory period was defined as the minimal S1-S2 coupling required to induce an AP which propagated to at least three-quarters of the distance down the strand (Whittaker et al., 2017).

The 3D left ventricular wedge model used (shown in Supplementary Figure S2) had spatial steps of Δ*x* = Δ*y* = 0.425 mm, and Δ*z* = 0.5 mm as determined by the DT-MRI dataset resolution, and incorporated fibre and sheet orientations (Benson et al., 2011). The value of **D** in an orthotropic configuration is thus dependent on the location within the ventricles. At each point in the ventricular mass, orthogonal vectors **f**, **s**, and **n** represent the three principal orthogonal directions of diffusion – along the fibre axis, perpendicular to the fibre axis in the sheet plane, and normal to the sheet plane. Scalar components of vectors **f**, **s**, and **n** with respect to a global Cartesian coordinate system are contained within the DT-MRI dataset. Based on these orthogonal vectors, the diffusion tensor at a given point in space in a local coordinate system is given by

$$\tilde{\text{D}}=\left( \begin{matrix} D_{||} & 0 & 0 \\ 0 & D_{\perp1} & 0 \\ 0 & 0 & D_{\perp2} \end{matrix} \right)$$

where *D*_||_ is diffusion in the fibre direction, *D*_⊥1_ is diffusion perpendicular to the fibre axis in the sheet plane, and D_⊥2_ is diffusion normal to the sheet plane (i.e. corresponding to diffusion in orthogonal directions **f**, **s**, and **n**, respectively). A transform of $\tilde{\text{D}}$ in the local coordinate system is required to find components of the diffusion tensor **D** in the global Cartesian coordinate system. As the orthogonal vectors **f**, **s**, and **n** are eigenvectors of the diffusion coefficient **D** with corresponding eigenvalues *D*_||_, *D*_⊥1_, and D_⊥2_, the orthogonal matrix **A** = (**f**, **s**, **n**) is used as the transformation matrix. The diffusion tensor **D** in the global Cartesian coordinate system is given by $\text{D}=\text{A}\tilde{\text{D}}\text{A}^{\text{T}}$ where T is the transpose of the matrix. An entry in **D** is thus given by

$\text{D}=D_{||}\text{f}\text{f}^{\text{T}}\boldsymbol{+}D_{\boldsymbol{\perp}1}\text{s}\text{s}^{\text{T}}\boldsymbol{+}D_{\boldsymbol{\perp}2}\text{n}\text{n}^{\text{T}}$**.**

The value of *D*_||_ was set to 0.1171 mm^2^ms^-1^ in this study (Bueno-Orovio et al., 2008), with anisotropic and orthotropic conductivity ratios of *D*_||_:*D*_⊥1_ = 4:1 and *D*_||_:*D*_⊥2_ = 9:1, respectively (Benson et al., 2011). Further details regarding development and validation of the 3D ventricular wedge geometry can be found in (Benson et al., 2011). In addition, details on the phase distribution method, which was used as an efficient method for initiating re-entrant waves in the 3D wedge in this study, can be found in previous studies (Biktashev and Holden, 1998; Colman et al., 2017; Whittaker et al., 2017).

# Supplementary Figures


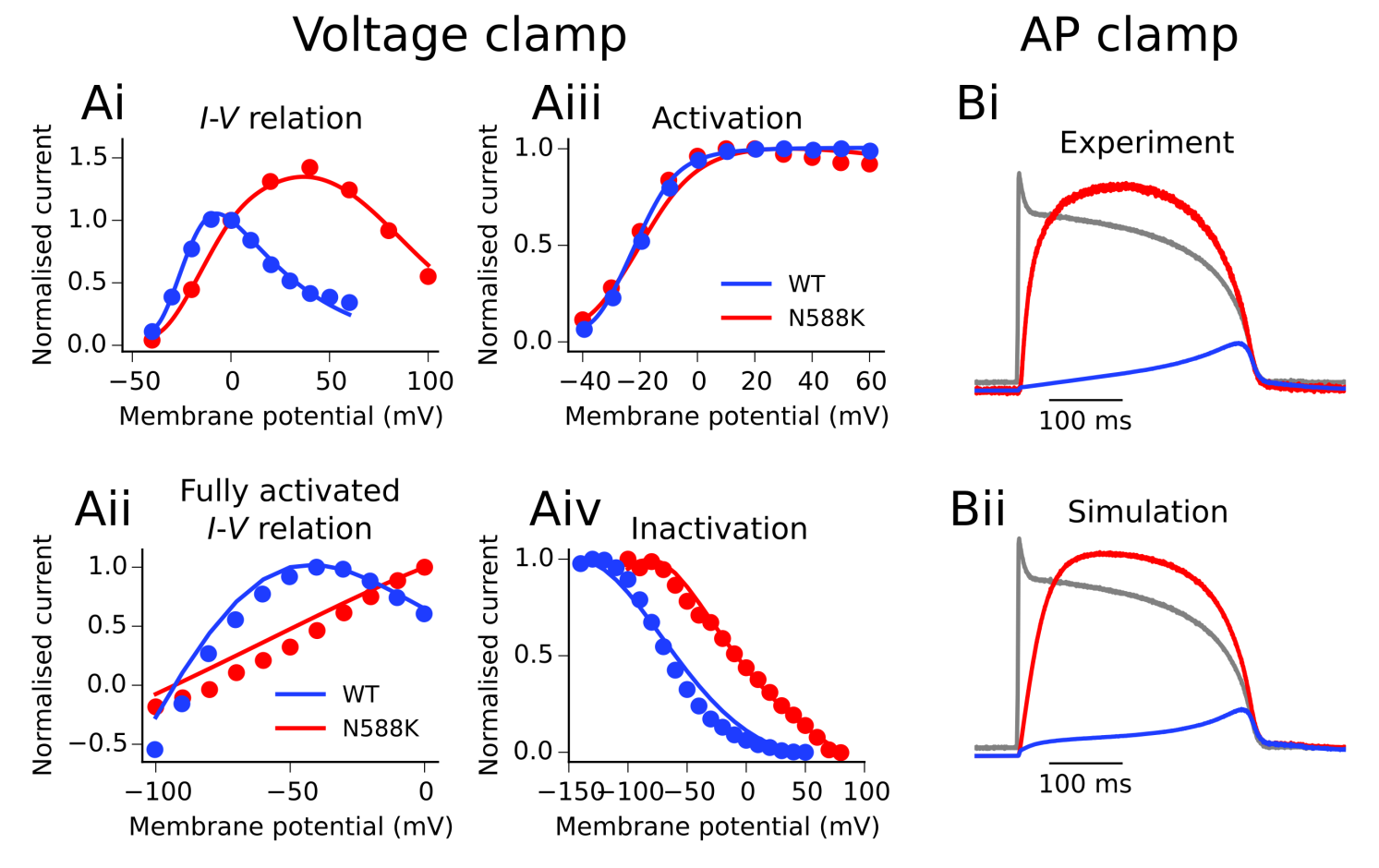
Figure S1: Experimental validation of new Markov chain models of WT and SQT1-N588K mutant *I*_Kr_. Voltage clamp experimental protocol simulations (solid lines) for the *I*-*V* relation (Ai), fully activated *I*-*V* relation (Aii), steady state activation (Aiii) and inactivation (Aiv) are compared to experimental data (points) (McPate et al., 2005) for WT (blue) and N588K (red) conditions. Experimental (Bi) and simulated (Bii) action potential (AP) clamps using a simulated human ventricular AP waveform (McPate et al., 2009).


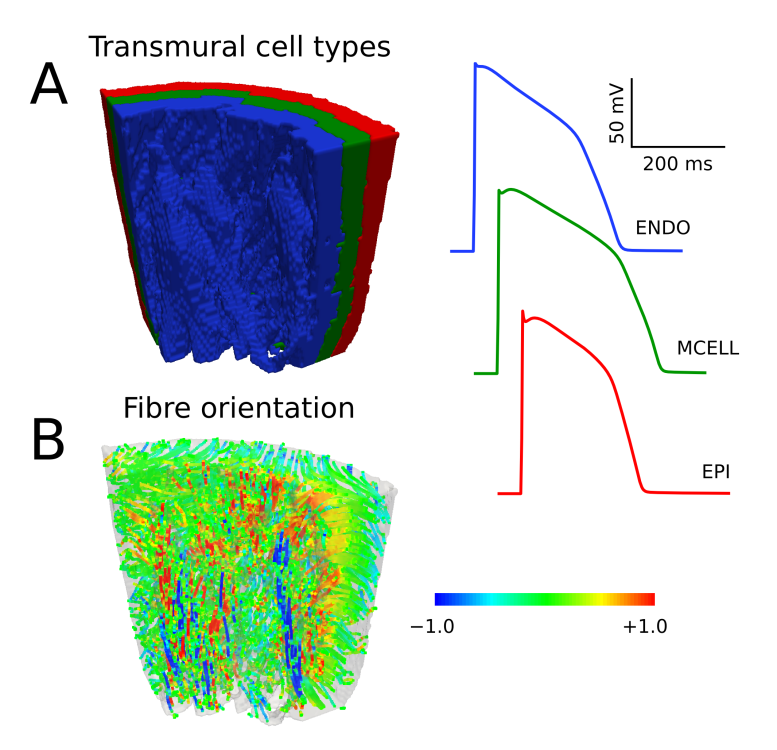


Figure S2: The 3D left ventricular wedge with segmented regions and traced fibre orientations showing the microstructure of the ventricular wall. (A) Segmented transmural regions in the ventricular wedge from an endocardial aspect, with corresponding transmural cellular endocardial (ENDO - blue), mid-myocardial (MCELL – green), and epicardial (EPI – red) action potentials. (B) Traced fibre orientations. The colour map corresponds to the *z* component (vertical direction) of the longitudinal vector of the fibres (Benson et al., 2011).


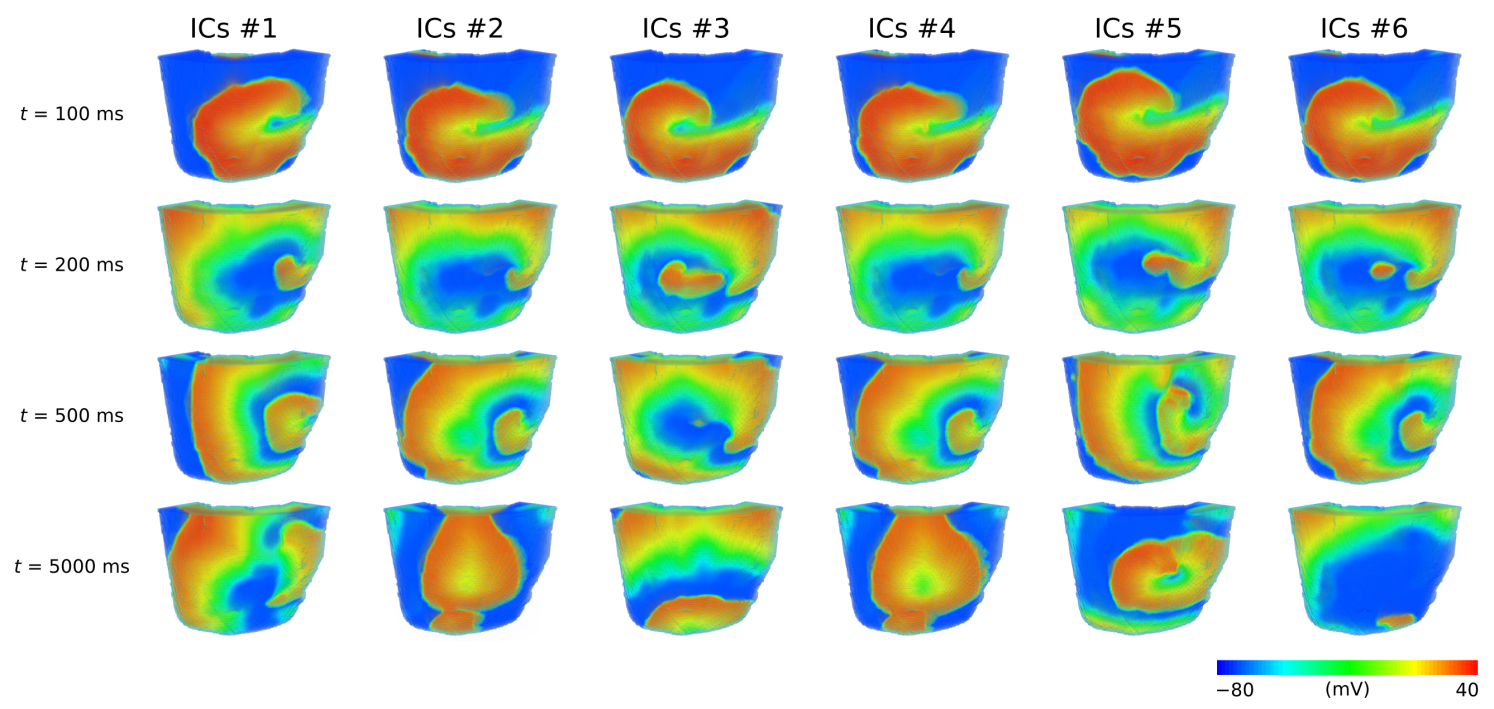
Figure S3: Development of scroll waves from different phase distribution initial conditions (*n* = 6). Snapshots showing the development of re-entrant scroll waves from *t* = 100 ms to *t* = 5000 ms using six different phase distribution initial conditions (ICs) in the 3D human left ventricular wedge geometry (Benson et al., 2011).


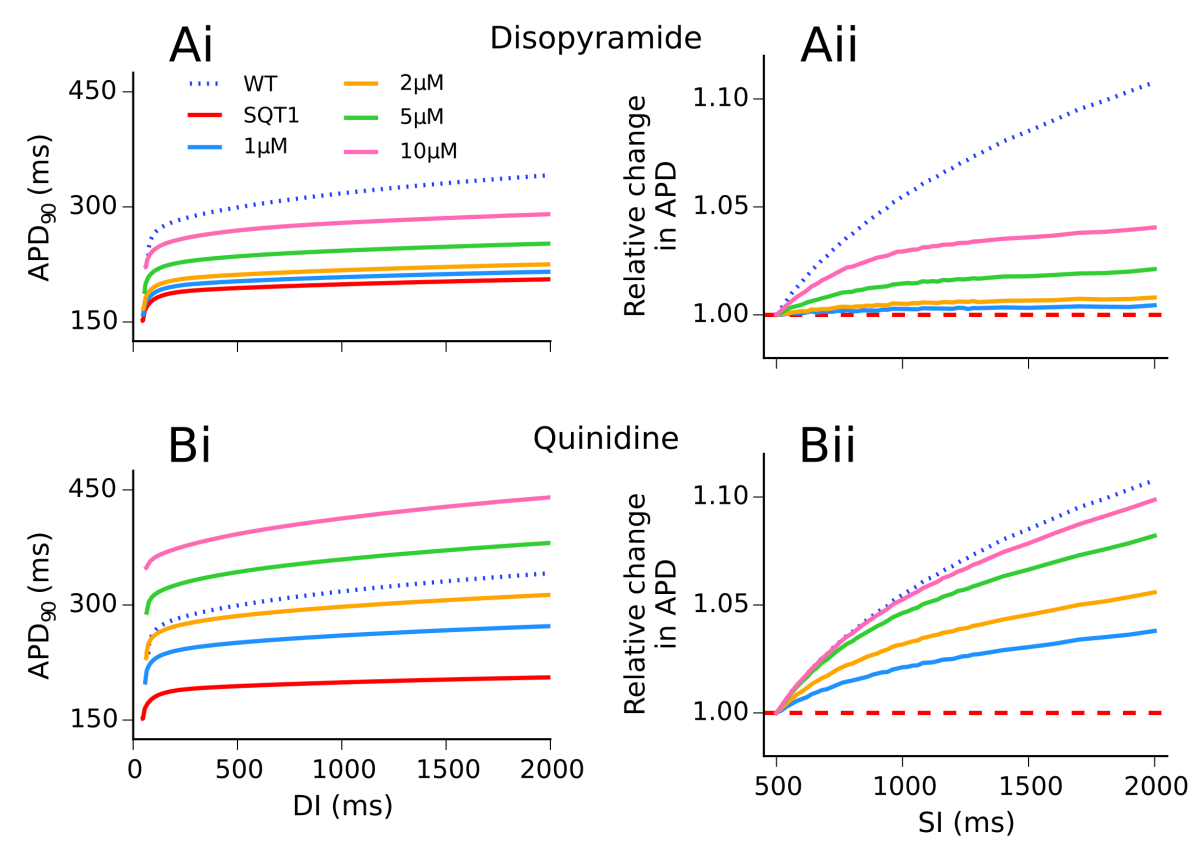


Figure S4: Restoration of rate adaptation by disopyramide and quinidine in the setting of SQT1. Restitution curves showing the APD as a function of diastolic interval (DI) computed using an S1-S2 protocol (S1 = 1000 ms) for drug-free WT and SQT1 conditions, as well as SQT1 + various concentrations of disopyramide (Ai) and quinidine (Bi). The relative change in APD, normalised with respect to the SQT1 APD at a stimulus interval (SI) of 500 ms for disopyramide (Aii) and quinidine (Bii).


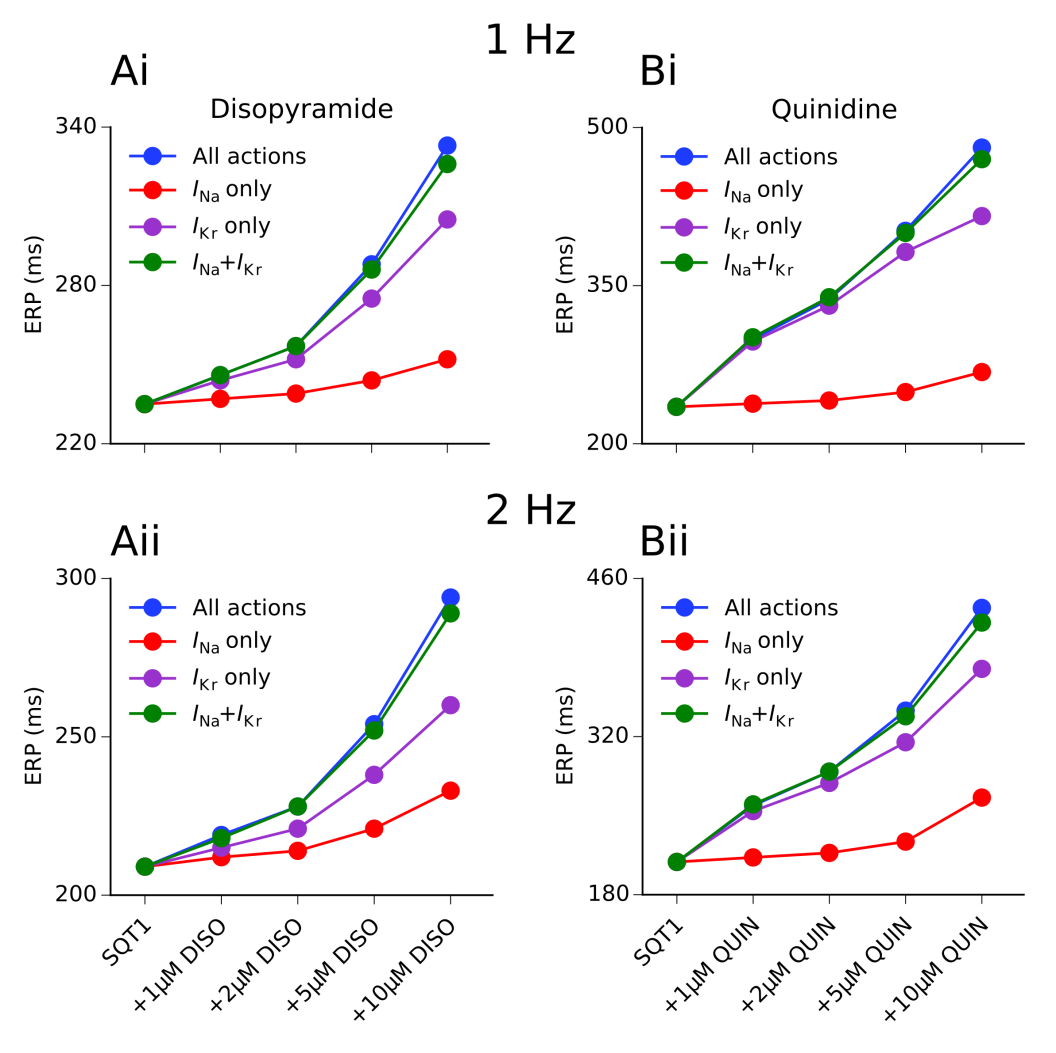


Figure S5: Rate-dependent ionic contributions of *I*_Kr_ and *I*_Na_ to ERP proplongation by disopyramide and quinidine. The dose-dependent increase in the effective refractory period (ERP) is shown for disopyramide (DISO) at pacing rates of 1 Hz (Ai) and 2 Hz (Aii) and quinidine (QUIN) at pacing rates of 1 Hz (Bi) and 2 Hz (Bii) for the following scenarios: all combined actions (blue), actions on *I*_Na_ only (red), actions on *I*_Kr_ only (purple), and combined actions on *I*_Na_ and *I*_Kr_ only (green).


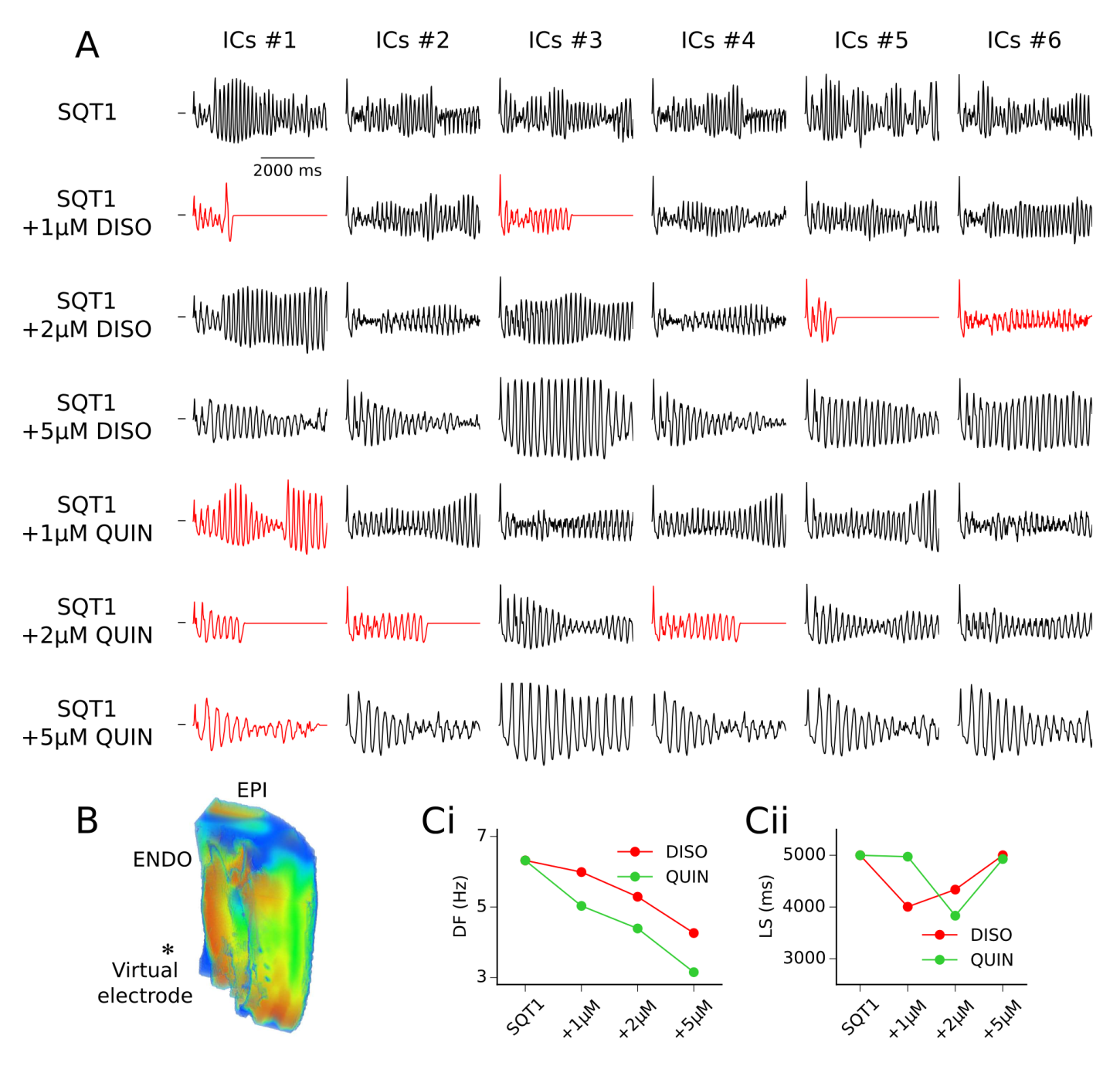


Figure S6: A summary of pseudo ECG waveforms in 3D human ventricular wedge re-entry simulations. (A) Pseudo ECG (pECG) waveforms for the six different phase distribution initial conditions (ICs) shown in Supplementary Figure S2 for SQT1, and SQT1 + various concentrations of disopyramide (DISO) and quinidine (QUIN). Cases in which re-entrant activity terminated within the 5.0 s simulation period are highlighted in red. (B) The location of the virtual electrode used for calculation of the pECG is shown relative to the wedge geometry, with endocardial (ENDO) and epicardial (EPI) surfaces labelled for reference. Averaged values of the dominant frequency (DF) (Ci) and lifespan (LS) of re-entry (Cii) are summarised.


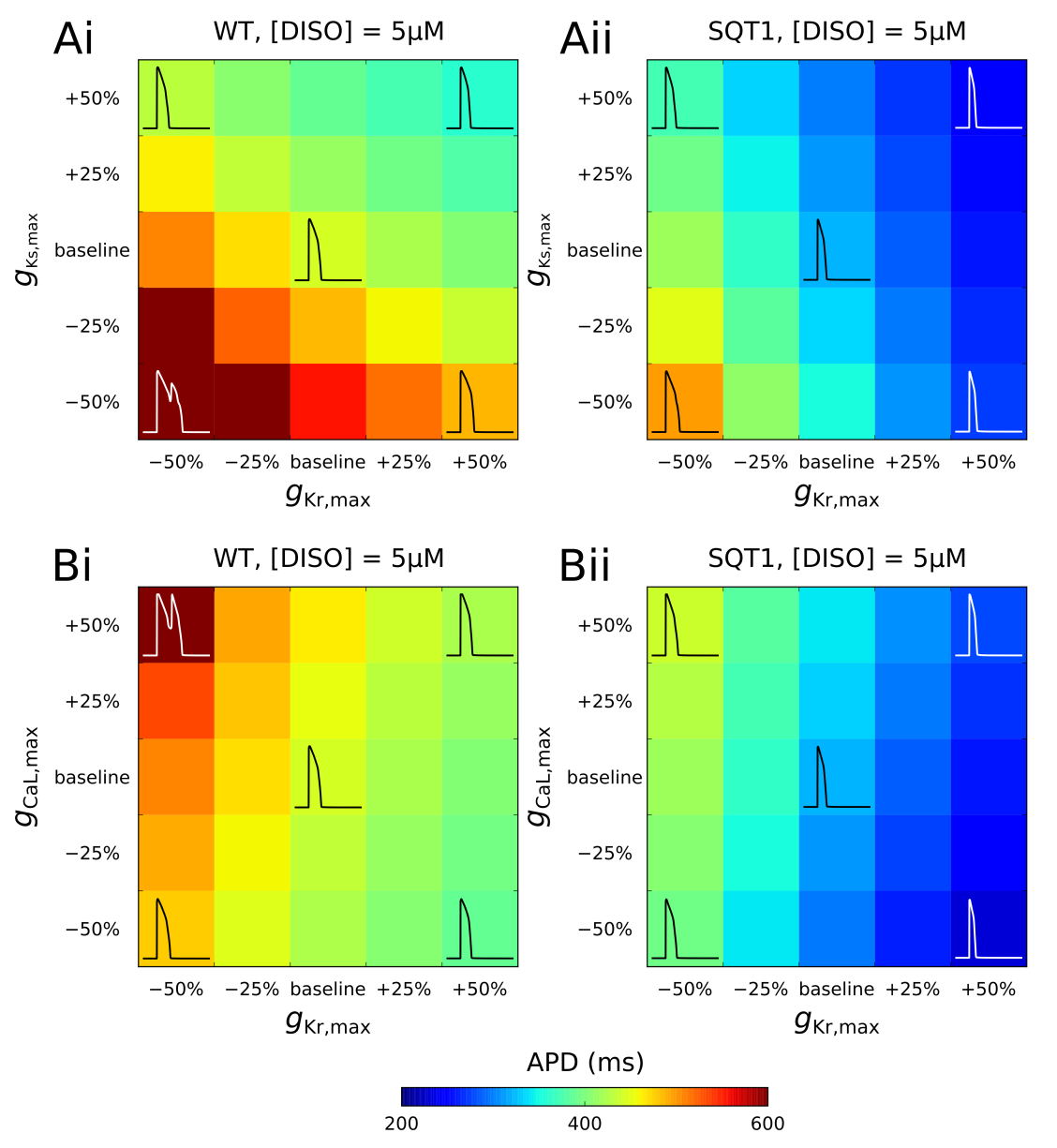


Figure S7: Screening for development of early after-depolarisations upon application of 5 μM disopyramide under variant parameter combinations. The effects of parameter variations in the *g*_Ks_-*g*_Kr_ parameter space on development of early after-depolarisations (EADs) following application of 5 μM disopyramide (DISO) in WT (Ai) and SQT1 (Aii) conditions. The effects of parameter variations in the *g*_CaL_-*g*_Kr_ parameter space on development of EADs following application of 5 μM DISO in WT (Bi) and SQT1 (Bii) conditions. Representative APs are shown in the centre and corner panels of the grid.


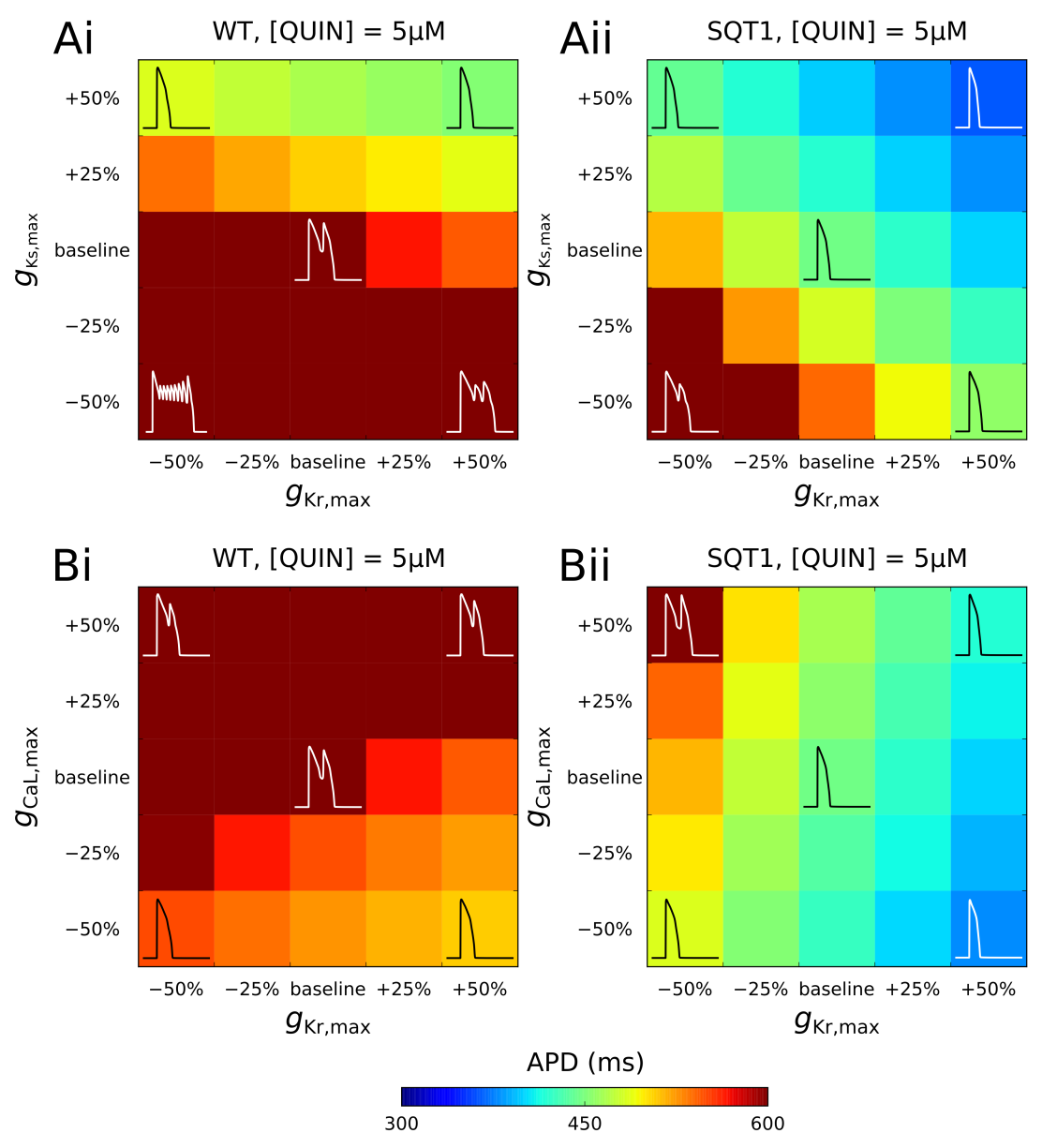


Figure S8: Screening for development of early after-depolarisations upon application of 5 μM quinidine under variant parameter combinations. The effects of parameter variations in the *g*_Ks_-*g*_Kr_ parameter space on development of early after-depolarisations (EADs) following application of 5 μM quinidine (QUIN) in WT (Ai) and SQT1 (Aii) conditions. The effects of parameter variations in the *g*_CaL_-*g*_Kr_ parameter space on development of EADs following application of 5 μM QUIN in WT (Bi) and SQT1 (Bii) conditions. Representative APs are shown in the centre and corner panels of the grid.


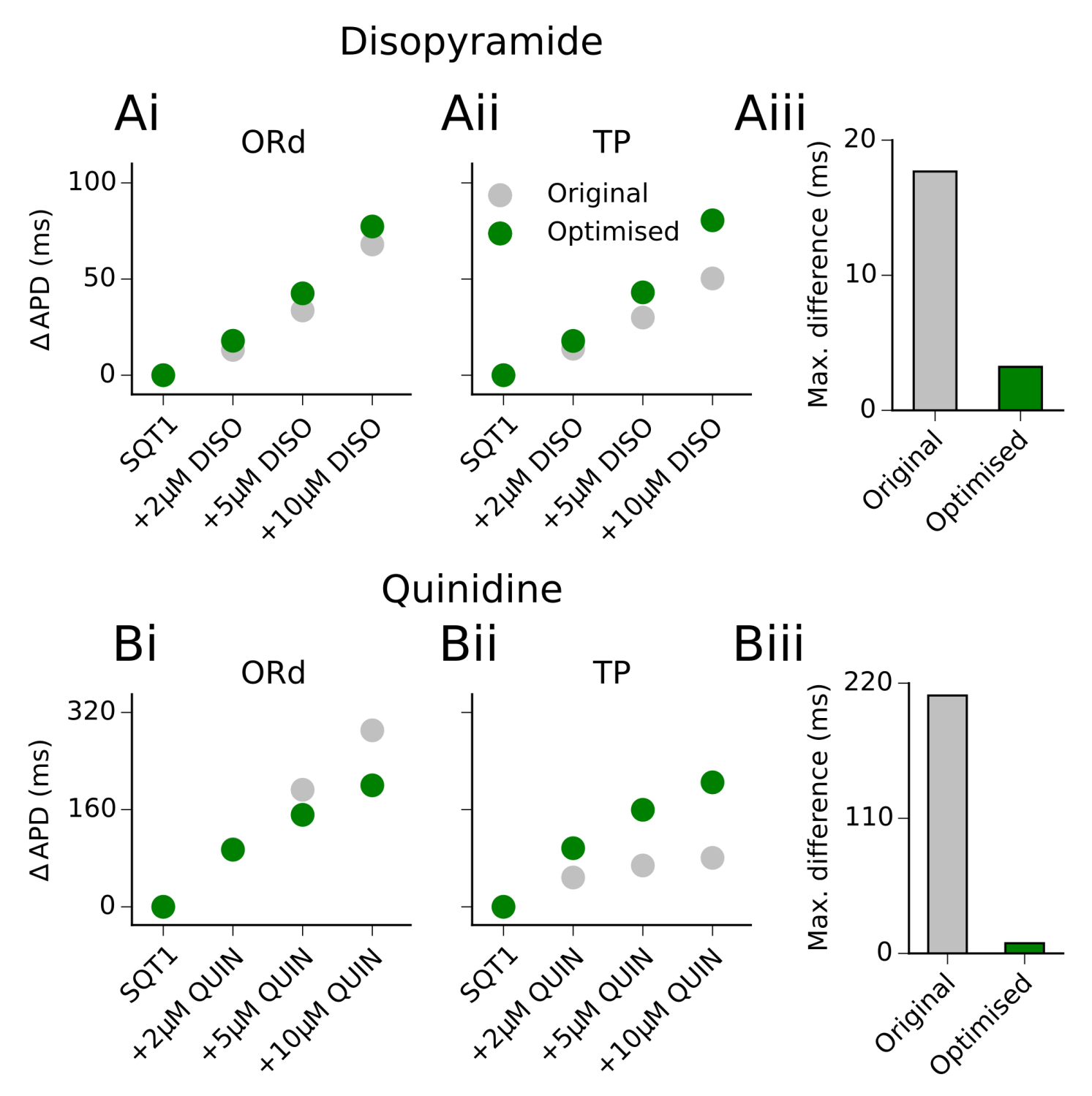


Figure S9: Prolongation of the endocardial AP in the ORd and TP models upon application of disopyramide and quinidine. Action potential prolongation, ΔAPD, in original (grey) and optimised (green) forms of (i) the ORd model (O’Hara et al., 2011) and (ii) the TP model (Tusscher and Panfilov, 2006) of human ventricular myocytes under application of varying concentrations of disopyramide (A) and quinidine (B). In each case the maximum difference between ORd and TP model outputs for all doses is shown for original and optimised forms of the models (iii). All simulations performed in endocardial cells at 1 Hz. Optimised forms of models are described in (Mann et al., 2016).

**
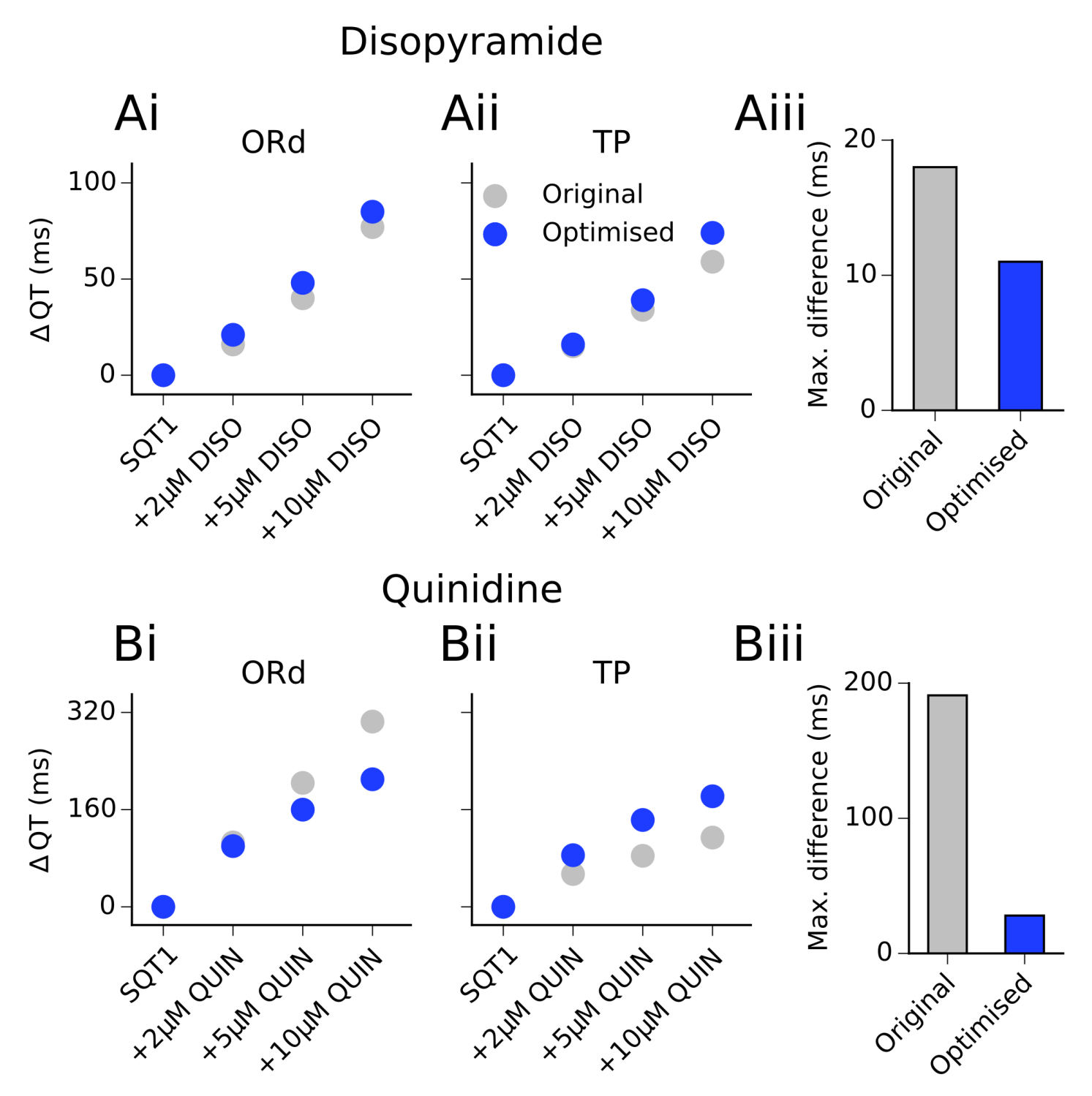
**

Figure S10: Prolongation of the QT interval in the ORd and TP models upon application of disopyramide and quinidine. QT interval prolongation, ΔQT, in original (grey) and optimised (blue) forms of (i) the ORd and (ii) the TP model under application of varying concentrations of disopyramide (A) and quinidine (B). In each case the maximum difference between model outputs for all doses is shown for original and optimised forms of the models (iii). All simulations performed in 1D strand models, as described in the manuscript, at 1 Hz.

**
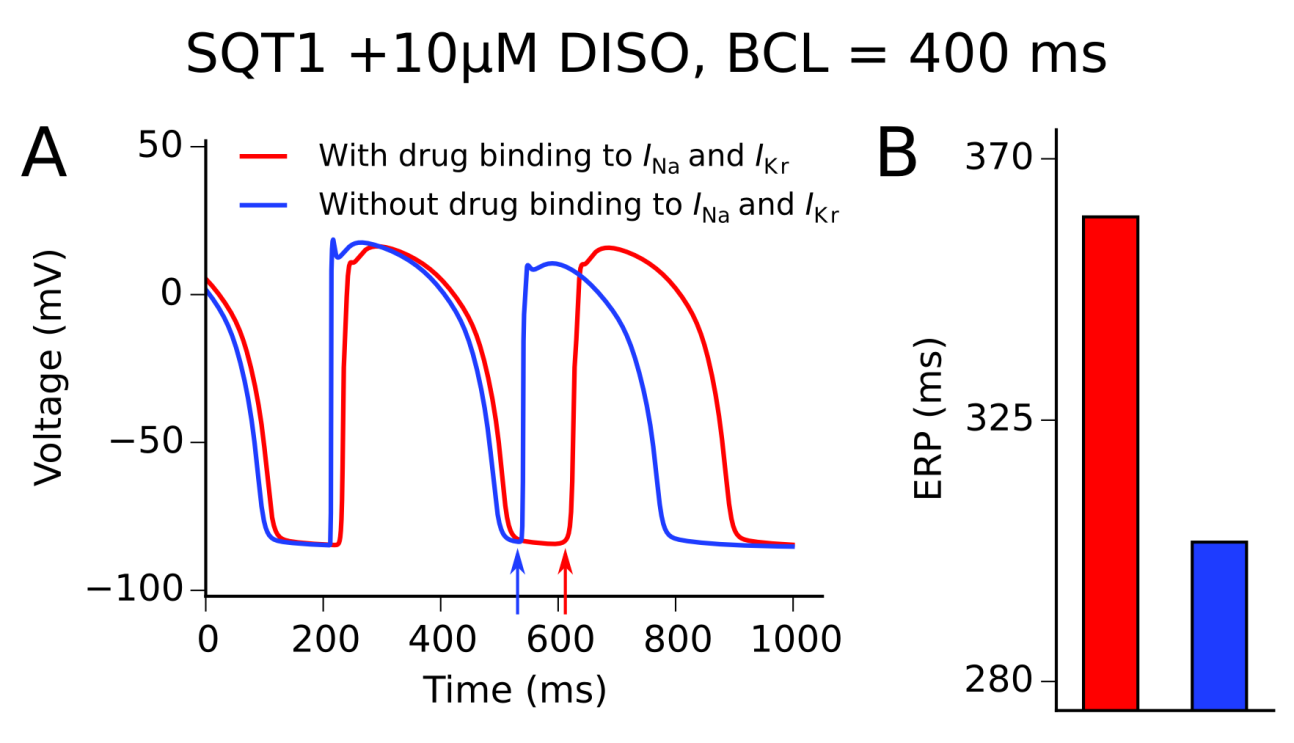
**

Figure S11: Effective refractory period in the TP model at a fast pacing rate both with and without drug binding to *I*_Na_ and *I*_Kr_. (A) Action potentials extracted from the centre of a 1D strand are shown both when drug binding to *I*_Na_ and *I*_Kr_ is considered (red) and when simple pore blocks of *I*_Na_ and *I*_Kr_ are used (blue). An S1-S2 protocol was employed with an S1 basic cycle length (BCL) of 400 ms, and S2 stimulus timing which is illustrated with an arrow in each case. (B) The corresponding effective refractory period (ERP) is shown.

# Supplementary Tables

**Table S1: *I*_Kr_ Markov chain parameters in WT and SQT1-N588K mutant conditions.** Transition rates corresponding to the MC scheme shown in manuscript Figure 1 are presented.

|  | **WT** | **N588K** |
| --- | --- | --- |
| ***α*** | $1.8\cdot{10}^{-1}\cdot exp(4.6\cdot{10}^{-2}\cdot\left( V-2.6\cdot{10}^{1} \right))$ | $1.1\cdot{10}^{-1}\cdot exp(2.2\cdot{10}^{-2}\cdot\left( V-3.5\cdot{10}^{1} \right))$ |
| ***β*** | $1.9\cdot{10}^{-1}\cdot exp(-4.6\cdot{10}^{-2}\cdot\left( V \right))$ | $7.1\cdot{10}^{-2}\cdot exp(-6.3\cdot{10}^{-2}\cdot\left( V \right))$ |
| ***α*_1_** | $2.7\cdot{10}^{0}$ | $3.8\cdot{10}^{0}$ |
| ***β*_1_** | $1.6\cdot{10}^{0}$ | $1.0\cdot{10}^{1}$ |
| ***α*_2_** | $7.4\cdot{10}^{-2}\cdot exp(1.8\cdot{10}^{-2}\cdot\left( V-7.6\cdot{10}^{1} \right))$ | $8.9\cdot{10}^{-2}\cdot exp(1.9\cdot{10}^{-2}\cdot\left( V-2.4\cdot{10}^{1} \right))$ |
| ***β*_2_** | $2.7\cdot{10}^{-3}\cdot exp(-6.5\cdot{10}^{-3}\cdot\left( V \right))$ | $2.1\cdot{10}^{-3}\cdot exp(-2.5\cdot{10}^{-5}\cdot\left( V \right))$ |
| ***α*_i_** | $1.7\cdot{10}^{-1}\cdot exp(-1.7\cdot{10}^{-2}\cdot(V+1.6\cdot{10}^{1})\cdot\left( {4.5}/{K_{o}} \right))$ | $2.8\cdot{10}^{-1}\cdot exp(-1.8\cdot{10}^{-2}\cdot\left( V+4.4\cdot{10}^{1} \right)\cdot\left( {4.5}/{K_{o}} \right))$ |
| ***β*_i_** | $8.5\cdot{10}^{-1}\cdot exp(1.8\cdot{10}^{-2}\cdot(V)\cdot\left( {4.5}/{K_{o}} \right)^{0.3})$ | $1.2\cdot{10}^{-2}\cdot exp(1.8\cdot{10}^{-2}\cdot(V)\cdot\left( {4.5}/{K_{o}} \right)^{0.3}$) |
| ***μ*** | $\left( \alpha_{i}\cdot\beta_{2} \right)/\beta_{i}$ | $\left( \alpha_{i}\cdot\beta_{2} \right)/\beta_{i}$ |

**Table S2: Rate transitions to drug-bound states of the Markov chain scheme for *I*_Kr_.** Binding (*k*) and unbinding (*l*) rates to drug-bound activated (A) and inactivated (I) states corresponding to the drug-bound Markov chain scheme presented in manuscript Figure 1. Drug affinities for states of type X were determined by computing *l*_X_/*k*_X_.

|  | **Disopyramide** | **Quinidine** |
| --- | --- | --- |
| ***k*_A_ (μM^-1^s^-1^)** | 2.01×10^1^ | 1.44×10^3^ |
| ***l*_A_ (s^-1^)** | 2.87×10^-4^ | 4.37×10^-3^ |
| ***k*_I_ (μM^-1^s^-1^)** | 7.14×10^-1^ | 1.91×10^2^ |
| ***l*_I_ (s^-1^)** | 8.99×10^-6^ | 1.29×10^-5^ |
| **Open state affinity (μM)** | 1.43×10^1^ | 3.02×10^0^ |
| **Inactivated state affinity (μM)** | 1.26×10^1^ | 6.75×10^-1^ |

**Table S3: Rate transitions for the guarded receptor model of drug binding to *I*_Na_.** Binding (*k*) and unbinding (*l*) rates to drug-bound activated (A), inactivated (I), and resting (R) states corresponding to the guarded receptor model equations presented in the manuscript. Drug affinities for states of type X were determined by computing *l*_X_/*k*_X_.

|  | **Disopyramide** | **Quinidine** |
| --- | --- | --- |
| ***k*_A_ (μM^-1^s^-1^)** | 9.40×10^-1^ | 1.87×10^0^ |
| ***l*_A_ (s^-1^)** | 1.70×10^-7^ | 1.73×10^-7^ |
| ***k*_I_ (μM^-1^s^-1^)** | 5.63×10^-1^ | 8.90×10^-1^ |
| ***l*_I_ (s^-1^)** | 1.07×10^-6^ | 1.12×10^-6^ |
| ***k*_R_ (μM^-1^s^-1^)** | 2.09×10^-1^ | 2.58×10^-1^ |
| ***l*_R_ (s^-1^)** | 8.05×10^-6^ | 4.60×10^-6^ |
| **Open state affinity (μM)** | 1.81×10^-1^ | 9.23×10^-2^ |
| **Inactivated state affinity (μM)** | 1.89×10^0^ | 1.26×10^0^ |
| **Resting state affinity (μM)** | 3.84×10^1^ | 1.78×10^1^ |

**Table S4: A comparison of simulated and clinically-observed QT interval increases due to application of disopyramide and quinidine.** A summary of simulated (shaded blue) pseudo ECG QT interval increase measured using a 1D strand model with application of various concentrations of disopyramide (DISO) and quinidine (QUIN) with clinical observations (shaded red), taken from genotyped-SQT1 patients where possible. HQ = hydroquinidine.

| **Disopyramide** | | | | | | | |
| --- | --- | --- | --- | --- | --- | --- | --- |
| **Simulation data** | | | **Clinical data** | | | | |
| **SQT1** | **SQT1 + DISO** | **% increase** | **SQTS** | **SQTS + DISO** | **% increase** | **Source** | **Notes** |
| 241 | 252 (1μM) | 4.6 | 329 | 358 | 8.8 | (Schimpf et al., 2007) | Patient 1, SQT1 |
|  | 262 (2μM) | 8.7 | 315 | 333 | 5.7 | (Schimpf et al., 2007) | Patient 2, SQT1 |
|  | 289 (5μM) | 19.9 | 327 ± 12 | 352 ± 17 | 7.6 ± 6.7 | (Giustetto et al., 2011) | 2 hERG, 1 non-hERG patient  (*n* =3) |
|  | 326 (10μM) | 35.2 | 306 | 424 | 38.6 | (Mizobuchi et al., 2008) | Genotype unknown |
| **Quinidine** | | | | | | | |
| **Simulation data** | | | **Clinical data** | | | | |
| **SQT1** | **SQT1 + QUIN** | **% increase** | **SQTS** | **SQTS + QUIN** | **% increase** | **Source** | **Notes** |
| 241 | 305 (1μM) | 26.6 | 263 ± 12 | 362 ± 25 | 37.6 ± 11.4 | (Gaita et al., 2004) | HQ, genotype unknown (*n* = 6) |
|  | 341 (2μM) | 41.5 | 299 ± 23 | 404 ± 30 | 35.1 ± 14.4 | (Giustetto et al., 2011) | HQ, SQT1  (*n* = 8) |
|  | 401 (5μM) | 66.4 | 289 ± 14 | 405 ± 26 | 40.1 ± 11.3 | (Giustetto, 2006) | HQ, SQT1  (*n* = 5) |
|  | 451 (10μM) | 87.1 | 306 ± 27 | 380 ± 43 | 24.2 ± 17.8 | (Frea et al., 2015) | HQ, only KCNH2  (*n* = 7) |

# Supplementary Videos

**Video S1: SQT1 re-entry in 3D human left ventricular wedge.** A representative video of initiation and conduction of re-entrant scroll waves in the drug-free SQT1 condition in the 3D human left ventricular wedge, shown from an epicardial aspect. The initiated scroll wave meanders unpredictably before degenerating into multiple, regenerative wavelets. Re-entrant activity sustains for the 5000 ms duration of the simulation.

**Video S2: SQT1 + 1μM disopyramide re-entry in 3D human left ventricular wedge.** A representative video of initiation and conduction of re-entrant scroll waves in the SQT1 condition with application of 1μM disopyramide in the 3D human left ventricular wedge, shown from an epicardial aspect. The initiated scroll wave forms a re-entrant circuit near the right boundary of the geometry before self-terminating at around *t* = 1360 ms.

**Video S3: SQT1 + 5μM disopyramide re-entry in 3D human left ventricular wedge.** A representative video of initiation and conduction of re-entrant scroll waves in the SQT1 condition with application of 5μM disopyramide in the 3D human left ventricular wedge, shown from an epicardial aspect. The initiated re-etrant excitation persists as a slowly-rotating scroll wave which drifts towards the lower boundary of the geometry, and sustains for the 5000 ms duration of the simulation.

**Video S4: SQT1 + 1μM quinidine re-entry in 3D human left ventricular wedge.** A representative video of initiation and conduction of re-entrant scroll waves in the SQT1 condition with application of 1μM quinidine in the 3D human left ventricular wedge, shown from an epicardial aspect. The initiated scroll wave forms a re-entrant circuit near the right boundary of the geometry which is destabilised by transient wave breaks. Re-entrant activity eventually self-terminates at around *t* = 4840 ms.

**Video S5: SQT1 + 5μM quinidine re-entry in 3D human left ventricular wedge.** A representative video of initiation and conduction of re-entrant scroll waves in the SQT1 condition with application of 5μM quinidine in the 3D human left ventricular wedge, shown from an epicardial aspect. The initiated re-entrant excitation persists as a slowly-rotating scroll wave which drifts towards the lower boundary of the geometry, and sustains for the 5000 ms duration of the simulation.

**References**

Adeniran, I., McPate, M. J., Witchel, H. J., Hancox, J. C., and Zhang, H. (2011). Increased Vulnerability of Human Ventricle to Re-entrant Excitation in hERG-linked Variant 1 Short QT Syndrome. *PLoS Comput. Biol.* 7. doi:10.1371/journal.pcbi.1002313.

Adeniran, I., Whittaker, D. G., Harchi, A. E., Hancox, J. C., and Zhang, H. (2017). In silico investigation of a KCNQ1 mutation associated with short QT syndrome. *Sci. Rep.* 7, 8469. doi:10.1038/s41598-017-08367-2.

Benson, A. P., Bernus, O., Dierckx, H., Gilbert, S. H., Greenwood, J. P., Holden, A. V., et al. (2011). Construction and validation of anisotropic and orthotropic ventricular geometries for quantitative predictive cardiac electrophysiology. *Interface Focus* 1, 101–116. doi:10.1098/rsfs.2010.0005.

Biktashev, V. N., and Holden, A. V. (1998). Reentrant waves and their elimination in a model of mammalian ventricular tissue. *Chaos Interdiscip. J. Nonlinear Sci.* 8, 48–56. doi:10.1063/1.166307.

Bueno-Orovio, A., Cherry, E. M., and Fenton, F. H. (2008). Minimal model for human ventricular action potentials in tissue. *J. Theor. Biol.* 253, 544–560. doi:10.1016/j.jtbi.2008.03.029.

Clayton, R. H., Bernus, O., Cherry, E. M., Dierckx, H., Fenton, F. H., Mirabella, L., et al. (2011). Models of cardiac tissue electrophysiology: Progress, challenges and open questions. *Prog. Biophys. Mol. Biol.* 104, 22–48. doi:10.1016/j.pbiomolbio.2010.05.008.

Colman, M. A., Ni, H., Liang, B., Schmitt, N., and Zhang, H. (2017). In silico assessment of genetic variation in KCNA5 reveals multiple mechanisms of human atrial arrhythmogenesis. *PLOS Comput. Biol.* 13, e1005587. doi:10.1371/journal.pcbi.1005587.

Drouin, E., Charpentier, F., Gauthier, C., Laurent, K., and Le Marec, H. (1995). Electrophysiologic characteristics of cells spanning the left ventricular wall of human heart: Evidence for presence of M cells. *J. Am. Coll. Cardiol.* 26, 185–192. doi:10.1016/0735-1097(95)00167-X.

Elshrif, M. M., and Cherry, E. M. (2014). A Quantitative Comparison of the Behavior of Human Ventricular Cardiac Electrophysiology Models in Tissue. *PLoS ONE* 9. doi:10.1371/journal.pone.0084401.

Frea, S., Giustetto, C., Capriolo, M., Scrocco, C., Fornengo, C., Benedetto, S., et al. (2015). New echocardiographic insights in short QT syndrome: More than a channelopathy? *Heart Rhythm* 12, 2096–2105. doi:10.1016/j.hrthm.2015.05.024.

Gaita, F., Giustetto, C., Bianchi, F., Schimpf, R., Haissaguerre, M., Calò, L., et al. (2004). Short QT syndrome: pharmacological treatment. *J. Am. Coll. Cardiol.* 43, 1494–1499. doi:10.1016/j.jacc.2004.02.034.

Gima, K., and Rudy, Y. (2002). Ionic Current Basis of Electrocardiographic Waveforms A Model Study. *Circ. Res.* 90, 889–896. doi:10.1161/01.RES.0000016960.61087.86.

Giustetto, C. (2006). “Quinidine to Treat Short QT Syndrome: A Real Alternative to ICD?,” in *Cardiac Arrhythmias 2005*, ed. A. R. MD (Springer Milan), 333–335. doi:10.1007/88-470-0371-7_42.

Giustetto, C., Schimpf, R., Mazzanti, A., Scrocco, C., Maury, P., Anttonen, O., et al. (2011). Long-Term Follow-Up of Patients With Short QT Syndrome. *J. Am. Coll. Cardiol.* 58, 587–595. doi:10.1016/j.jacc.2011.03.038.

Koumi, S., Sato, R., Katori, R., Hisatome, I., Nagasawa, K., and Hayakawa, H. (1992). Sodium channel states control binding and unbinding behaviour of antiarrhythmic drugs in cardiac myocytes from the guinea pig. *Cardiovasc. Res.* 26, 1199–1205. doi:10.1093/cvr/26.12.1199.

Li, G.-R., Feng, J., Yue, L., and Carrier, M. (1998). Transmural heterogeneity of action potentials andI to1 in myocytes isolated from the human right ventricle. *Am. J. Physiol. - Heart Circ. Physiol.* 275, H369–H377.

Luo, C. H., and Rudy, Y. (1994). A dynamic model of the cardiac ventricular action potential. I. Simulations of ionic currents and concentration changes. *Circ. Res.* 74, 1071–1096. doi:10.1161/01.RES.74.6.1071.

Mann, S. A., Imtiaz, M., Winbo, A., Rydberg, A., Perry, M. D., Couderc, J.-P., et al. (2016). Convergence of models of human ventricular myocyte electrophysiology after global optimization to recapitulate clinical long QT phenotypes. *J. Mol. Cell. Cardiol.* 100, 25–34. doi:10.1016/j.yjmcc.2016.09.011.

McPate, M. J., Duncan, R. S., Hancox, J. C., and Witchel, H. J. (2008). Pharmacology of the short QT syndrome N588K-hERG K+ channel mutation: differential impact on selected class I and class III antiarrhythmic drugs. *Br. J. Pharmacol.* 155, 957–966. doi:10.1038/bjp.2008.325.

McPate, M. J., Duncan, R. S., Milnes, J. T., Witchel, H. J., and Hancox, J. C. (2005). The N588K-HERG K+ channel mutation in the “short QT syndrome”: Mechanism of gain-in-function determined at 37 °C. *Biochem. Biophys. Res. Commun.* 334, 441–449. doi:10.1016/j.bbrc.2005.06.112.

McPate, M. J., Zhang, H., Adeniran, I., Cordeiro, J. M., Witchel, H. J., and Hancox, J. C. (2009). Comparative effects of the short QT N588K mutation at 37 degrees C on hERG K+ channel current during ventricular, Purkinje fibre and atrial action potentials: an action potential clamp study. *J. Physiol. Pharmacol. Off. J. Pol. Physiol. Soc.* 60, 23–41.

Mizobuchi, M., Enjoji, Y., Yamamoto, R., Ono, T., Funatsu, A., Kambayashi, D., et al. (2008). Nifekalant and Disopyramide in a Patient with Short QT Syndrome: Evaluation of Pharmacological Effects and Electrophysiological Properties. *Pacing Clin. Electrophysiol.* 31, 1229–1232. doi:10.1111/j.1540-8159.2008.01169.x.

Moreno, J. D., Lewis, T. J., and Clancy, C. E. (2016). Parameterization for In-Silico Modeling of Ion Channel Interactions with Drugs. *PLOS ONE* 11, e0150761. doi:10.1371/journal.pone.0150761.

O’Hara, T., Virág, L., Varró, A., and Rudy, Y. (2011). Simulation of the Undiseased Human Cardiac Ventricular Action Potential: Model Formulation and Experimental Validation. *PLoS Comput. Biol.* 7. doi:10.1371/journal.pcbi.1002061.

Paul, A. A., Witchel, H. J., and Hancox, J. C. (2001). Inhibition of HERG Potassium Channel Current by the Class 1a Antiarrhythmic Agent Disopyramide. *Biochem. Biophys. Res. Commun.* 280, 1243–1250. doi:10.1006/bbrc.2001.4269.

Paul, A. A., Witchel, H. J., and Hancox, J. C. (2002). Inhibition of the current of heterologously expressed HERG potassium channels by flecainide and comparison with quinidine, propafenone and lignocaine. *Br. J. Pharmacol.* 136, 717–729. doi:10.1038/sj.bjp.0704784.

Schimpf, R., Veltmann, C., Giustetto, C., Gaita, F., Borggrefe, M., and Wolpert, C. (2007). In vivo Effects of Mutant HERG K+ Channel Inhibition by Disopyramide in Patients with a Short QT-1 Syndrome: A Pilot Study. *J. Cardiovasc. Electrophysiol.* 18, 1157–1160. doi:10.1111/j.1540-8167.2007.00925.x.

Schimpf, R., Wolpert, C., Gaita, F., Giustetto, C., and Borggrefe, M. (2005). Short QT syndrome. *Cardiovasc. Res.* 67, 357–366. doi:10.1016/j.cardiores.2005.03.026.

Tusscher, K. H. W. J. ten, and Panfilov, A. V. (2006). Alternans and spiral breakup in a human ventricular tissue model. *Am. J. Physiol. - Heart Circ. Physiol.* 291, H1088–H1100. doi:10.1152/ajpheart.00109.2006.

Whittaker, D. G., Ni, H., Harchi, A. E., Hancox, J. C., and Zhang, H. (2017). Atrial arrhythmogenicity of KCNJ2 mutations in short QT syndrome: Insights from virtual human atria. *PLOS Comput. Biol.* 13, e1005593. doi:10.1371/journal.pcbi.1005593.

Yan, G.-X., Shimizu, W., and Antzelevitch, C. (1998). Characteristics and Distribution of M Cells in Arterially Perfused Canine Left Ventricular Wedge Preparations. *Circulation* 98, 1921–1927. doi:10.1161/01.CIR.98.18.1921.
